# Are African households (not) leaving agriculture? Patterns of households’ income sources in rural Sub-Saharan Africa^☆^

**DOI:** 10.1016/j.foodpol.2016.09.018

**Published:** 2017-02

**Authors:** Benjamin Davis, Stefania Di Giuseppe, Alberto Zezza

**Affiliations:** a Food and Agriculture Organization of the United Nations, Italy; b Universitá di Teramo and Food and Agriculture Organization of the United Nations, Italy; c World Bank, United States

**Keywords:** Income, Non-farm employment, Agriculture, Africa, LSMS

## Abstract

This paper uses comparable income aggregates from 41 national household surveys from 22 countries to explore the patterns of income generation among rural households in Sub-Saharan Africa, and to compare household income strategies in Sub-Saharan Africa with those in other regions. The paper seeks to understand how geography drives these strategies, focusing on the role of agricultural potential and distance to urban areas. Specialization in on-farm activities continues to be the norm in rural Africa, practiced by 52 percent of households (as opposed to 21 percent of households in other regions). Regardless of distance and integration in the urban context, when agro-climatic conditions are favorable, farming remains the occupation of choice for most households in the African countries for which the study has geographically explicit information. However, the paper finds no evidence that African households are on a different trajectory than households in other regions in terms of transitioning to non-agricultural based income strategies.

## 1 Introduction

Agriculture declines as a share of aggregate output with overall growth in GDP per capita as countries undergo the structural transformation that accompanies economic development (Chenery and Syrquin, 1975). In rural areas of developing countries, the decline in the relative importance of agriculture and the expansion of rural non-farm activities are likely features of the process of economic development. Growth in rural non-farm activities cannot be seen in isolation from agriculture, however, as both are linked through investment, production, and consumption throughout the rural economy, and in relation to urban centers, and both form part of complex livelihood strategies adopted by rural households. Better incentives for agriculture during the past decade, via the improvement of the policy environment and better terms of trade, provide a more conducive environment for higher agricultural growth and an opportunity for the much awaited structural transformation in Africa (Binswanger-Mkhize et al., 2010).

A rather large body of literature has developed over the last 20 years investigating the importance and features of rural non-farm income and employment in the developing world, the determinants of households’ participation in and returns to different income-generating activities, and the extent and determinants of rural household income diversification (FAO, 1998, Barrett et al., 2001, Lanjouw and Lanjouw, 2001, Haggblade et al., 2007, Winters et al., 2009, Winters et al., 2010, Davis et al., 2010). The 2007 World Development Report on agriculture and the 2011 IFAD Rural Poverty Report also devoted much attention to these themes. A major conclusion of these studies is that rural household income diversification is the norm rather than the exception, and that while endowments (e.g. physical, human, natural capital) and wealth play a role in driving engagement in different economic activities, some degree of diversification off the farm is common at all levels of welfare. Due to data limitations, however, the question remains as to whether this is occurring in Africa, a latecomer to the process of structural transformation. Conventional wisdom would have it that rural households in Sub-Saharan Africa are primarily employed in agriculture, with relatively little agricultural wage labor, and even less non-agricultural wage labor due to limited industrialization.

Less discussed in the literature is the role of geography in determining rural household income strategies. Deichmann et al. (2008) identify two main strands of literature that help frame the arguments around location and income diversification. First, one key empirical regularity of the rural farm/non-farm employment literature is that at very low levels of development, non-farm activities tend to be closely related to agriculture. Growth in the agricultural sector (e.g. due to technological change) leads to growth in the non-farm economy, thanks to the backward and forward linkages from agriculture.

Such growth patterns are not likely to be location neutral, as potential for agricultural growth and agro-industrial demand for agricultural products are not randomly allocated across space. Over time endogenous sectoral growth biases may play a role, as infrastructure and other investments may tend to locate where growth is occurring, leading to increased spatial disparities in growth patterns. In Latin America, this has attracted considerable attention in the context of the debate on the ‘territorial approach’ to rural development (de Ferranti et al., 2005). As sectoral policies are likely to have differential impacts across space, explicitly incorporating spatial issues into policy design can help counter territorial distortions in development patterns.

The second key strand of literature is the new economic geography debate, which focuses on the extent to which geography, as opposed to institutions, explains differential development outcomes. One main tenet of that debate is that even if soil quality and climate were the same everywhere, location would still matter. On the one hand, dispersion of economic activities occurs as firms tend to locate in areas with lower wages, and the production of non-tradable goods and services locates close to demand. Activities connected to non-mobile inputs (such as agricultural land) will by definition be spread across space to some extent. On the other hand, agglomeration pushes businesses to locate close to consumers or to the source of raw material. Businesses depending on mobile inputs but with higher transport costs for their outputs would tend to have the highest gains from concentrating in particular locations.

Moreover, the location of economic activities across space may be nonlinear. Fafchamps and Shilpi (2003) find for instance that in Nepal, agricultural wage employment is concentrated in rural areas close enough to cities to specialize in high-value horticulture, but not so close as to be taken over by unskilled ‘urban’ wage labor opportunities. Non-linearities may also be relevant when city size is found to matter for engagement in non-farm activities (Fafchamps and Shilpi, 2003) or for poverty reduction (Christiaensen et al., 2013). Also, specialization may be dependent upon a particular market size or specific types of markets (Fafchamps and Shilpi, 2005).

Agricultural potential and distance may interact in determining locational advantage, occupational choices and returns to economic activities, but relatively few empirical studies have been able to assess these interactions in low-income country settings. In Uganda, Yamano and Kijima (2010) show how soil fertility is positively associated with crop income, but not with non-farm income, whereas remoteness and poor road infrastructure lead to lower crop income. In Bangladesh, Deichmann et al. (2008) find that the higher the distance to an urban ‘growth pole’, the lower the level of employment in high-return non-farm jobs, particularly in areas with good agricultural potential.

Finally, different patterns of urbanization (megacities versus growth in small towns) may be associated with development outcomes, but the incentives and constraints driving them change with different stages of industrialization and urbanization processes, rendering them difficult for modeling. In early stages, resource-based industrialization may be geographically scattered, but as activities that are not based on natural resources increase, they tend to be located in large centers. The extent to which these activities will move to secondary urban centers and/or rural areas will depend upon the policy environment (Hamer and Linn, 1987).

Bringing these arguments and evidence together, it becomes clear that both exogenous physical location as well as the interaction between sectors and endogenous policy-related issues come into play in complex ways that complicate predictions of the spatial location of economic activities in rural areas.

Taking advantage of newly available data, this paper seeks to compare the income strategies of rural households in Sub-Saharan Africa with those of households in other countries, taking into account different levels of development. Specifically, this paper seeks to understand the role of agriculture in the rural economy, the profiles of households reducing their participation in the agricultural sector, and the degree to which income portfolio patterns can be linked to geographical features such as agro-ecological potential and urban access.

In order to answer these questions, we use comparable income aggregates from 41 national household surveys with high-quality income data conducted across 22 developing countries, constructed as part of FAO’s Rural Income Generating Activities (RIGA) project. The initial exploration of the RIGA database (Winters et al., 2009, Winters et al., 2010, Davis et al., 2010) highlighted a number of regularities concerning household patterns of income diversification in developing countries. The Sub-Saharan African countries included in the database stood out as the only countries for which specialization in farming, as opposed to holding a diversified income portfolio, was the norm.

That analysis was however based on data for only four countries in Sub-Saharan Africa: Madagascar, Malawi, Nigeria, Ghana. This paper takes advantage of more recent data from some of the same countries and additionally includes data on five more countries (Ethiopia, Kenya, Niger, Tanzania, Uganda), collected as part of the Living Standard Measurement Study - Integrated Surveys on Agriculture (LSMS-ISA)^1^ project. This new set of countries accounts for 51 percent of the Sub-Saharan African (SSA) population in 2012, as opposed to 26 percent in the initial RIGA sample. While caution is still warranted in treating this sample as representative of SSA as a whole, its coverage is arguably much more complete. Also, we take advantage of the geo-referencing of households and of the focus on agricultural activities that are two of the defining features of the LSMS-ISA datasets, in order to analyze the role of geography in shaping rural income strategies.

The paper continues as follows. In Section 2, we present and describe the construction of the RIGA database. In Section 3, we analyze the participation of rural households in income-generating activities and the share of income from each activity in household income, across all households and by expenditure quintile. We then move from the level of rural space to that of the rural household, examining patterns of diversification and specialization in rural income-generating activities, again across all households, and by expenditure quintile. We also use measures of stochastic dominance to characterize the relationship between types of income-generating strategies and welfare. In Section 4, we examine the role of location in income generation strategies in a multivariate framework, and we conclude in Section 5.

## 2 The data

### 2.1 The RIGA database

The RIGA database is constructed from a pool of several dozen Living Standards Measurement Study surveys (LSMSs) and from other multi-purpose household surveys made available by the World Bank through a joint project with the FAO.^2^ The most recent additions are the LSMS-ISA project countries (see complete list in Appendix Table A1). Each survey is representative for both urban and rural areas; only the rural sample was used for this paper.^3^ While clearly not representative of all developing countries, or all of Sub-Saharan Africa, the list does cover a significant range of countries, regions, and levels of development and has proven useful in providing insight into the income-generating activities of rural households in the developing world.^4^

Following Davis et al. (2010), income is classified into seven categories: (1) crop production; (2) livestock production; (3) agricultural wage employment, (4) non-agricultural wage employment; (5) non-agricultural self-employment; (6) transfer; and (7) other. 5 All income is net of input costs. Non-agricultural wage employment and non-agricultural self-employment income have been further disaggregated by industry using standard industrial codes, although we do not take advantage of this disaggregation in this study.

The seven income categories are aggregated into higher level groupings depending on the type of analysis. One grouping distinguishes between agricultural (i.e. crop, livestock, and agricultural wage income) and non-agricultural activities (i.e. non-agricultural wage, non-agricultural self-employment, transfer, and other income), and in a second, crop and livestock income are referred to as on-farm activities, non-agricultural wage and self-employment income as non-farm activities, and agricultural wage employment, transfer, and other income are left as separate categories. Finally, we also use the concept of off-farm activities, which includes all non-agricultural activities plus agricultural wage labor.

Income shares can be analyzed as the mean of income shares or as the share of mean income. In the first instance, income shares are calculated for each household, and then the mean of the household shares of each income category. In the second case, income shares are calculated as the share of a given source of income over a given group of households.^6^ Since the household is our basic unit of analysis, we use the mean of shares throughout this paper.

To analyze the spatial patterns of income generation, a set of geo-referenced variables from external sources are linked to the household-level data via their GPS attributes. This can only be done for the 6 LSMS-ISA datasets covering Ethiopia, Malawi, Niger, Nigeria, Tanzania, and Uganda. First, we use an aridity index as proxy for agricultural potential, which is defined as the ratio between mean annual precipitation and mean annual potential evapo-transpiration (thus, a higher value of the index identifies wetter areas).^7^ This is a purely physical, exogenous indicator that reflects long-term conditions in a locality. We maintain that this indicator is superior to alternatives that embed the profitability or value of agricultural production in a given area, as those incorporate contingent factors such as prices and terms of trade. In this application, we value the fact that the aridity index is truly exogenous.

Second, we proxy market access, distance and agglomeration effects with variables that measure the Euclidean (‘as the crow flies’) distance to cities of 20, 100, and 500 thousand inhabitants. We choose this measure due to a concern with the potential endogeneity of travel time measures; roads and travel infrastructure may be built in response to agricultural production or potential (Fafchamps and Shilpi, 2005, Deichmann et al., 2008). The Euclidean distance is independent of travel infrastructure, but provides a reliable measure of the spatial dispersion of households with regards to urban populations.

## 3 The diversity of income sources in Sub-Saharan Africa

### 3.1 Agriculture is still the main source of livelihoods in rural Sub-Saharan Africa

**Fig. 1:**
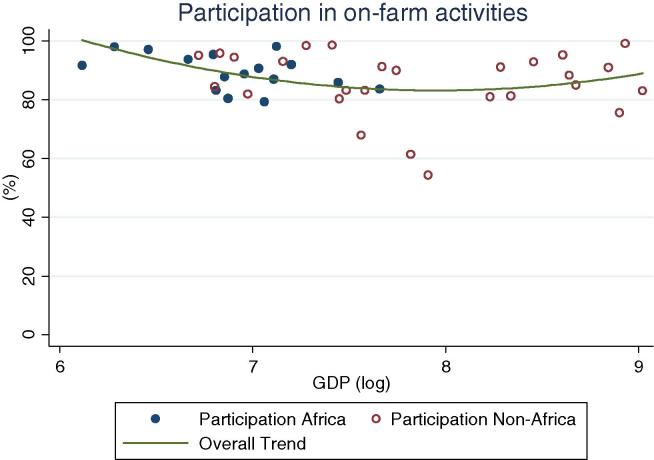
Percentage of rural households participating in on farm activities, by per capita GDP in 2005 PPP dollars.

**Fig. 2:**
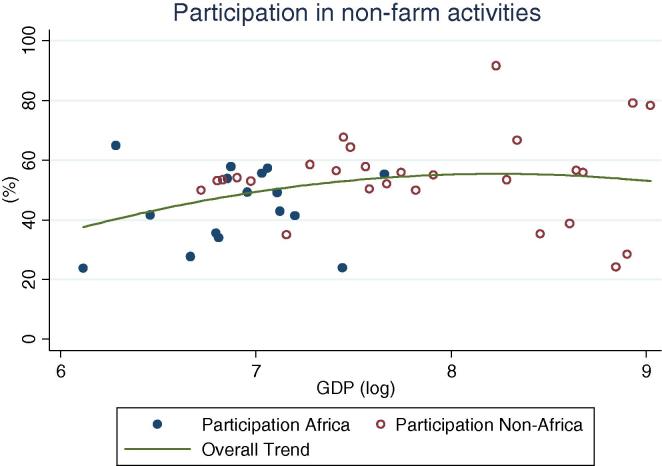
Percentage of rural households participating in non-farm activities, by per capita GDP in 2005 PPP dollars.

**Fig. 3:**
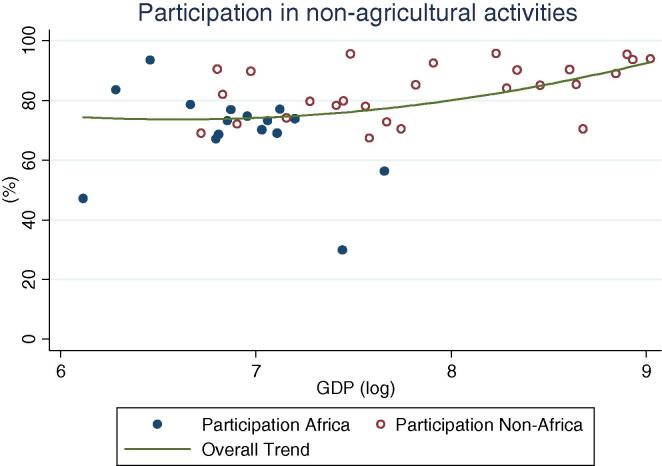
Percentage of rural households participating in non-agricultural activities, by per capita GDP in 2005 PPP dollars.

**Fig. 4:**
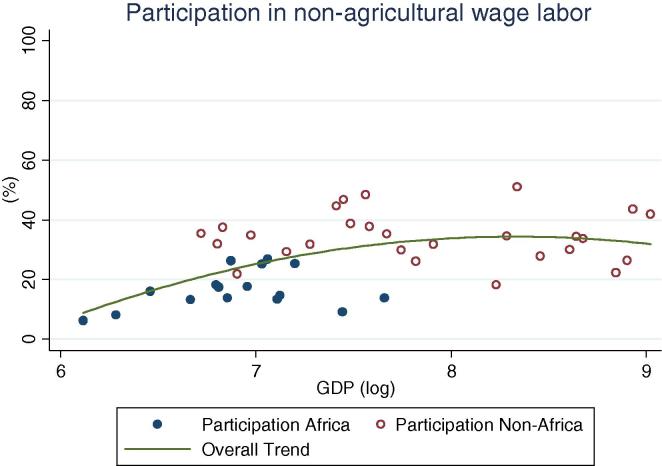
Percentage of rural households participating in non-agricultural wage labor, by per capita GDP in 2005 PPP dollars.

We begin by looking at the prevalence of household participation in different activities (Table 1, Fig. 1, Fig. 2, Fig. 3, Fig. 4).^8^ The discussion in this section is based on an analysis of the basic descriptive statistics, aided by a visual interpretation of scatterplots including simple quadratic trend lines fitted to the data.^9^ Strikingly, the near totality of rural households in the countries of our sample are engaged in own account agriculture. This is true in Africa (92 percent on average), but also in other regions (85 percent) (Fig. 1). While for some households the importance of this participation is relatively minor, since it includes consumption of a few animals or patio crop production, agriculture continues to play a fundamental role in the rural household economic portfolio. It is hard to overemphasize this result, especially given its robustness across countries and income levels: in the vast majority of the surveys we find that more than 8 in 10 rural households depend to some extent on agriculture. Regardless of the level of GDP, agriculture continues to be the distinctive feature of rural livelihoods.

At the same time, an important share of rural households, across GDP levels, participate in non-farm (non-agricultural wage labor and self-employment, Fig. 2). Globally, shares vary widely, ranging from 24 percent (Ethiopia and Nigeria 2004) to over 90 percent (Bolivia 2005). The simple mean non-farm participation share for African countries is 44 percent, which is 10 percentage points lower than for non-African countries. Among African countries, the highest share is observed in Niger, at 65 percent. A similar share of households obtains income from public or private transfer income, although it spans an even wider range, from 3 percent of households in Nigeria in 2010 to almost 90 percent in Malawi in 2004. When including non-farm, transfers and other sources of income, the vast majority of rural households across GDP levels have some form of off-farm income (see last column in Table 1), with rates higher in other regions (91 percent on average) than in Africa (74 percent). Participation in non-agricultural wage labor, on the other hand, shows a clear increase by levels of GDP (Fig. 4), with the African countries in our sample (shown in blue or darker hue) reporting relatively lower participation rates (from 2 percent in Ethiopia to 25 percent in Kenya and Uganda 2009/10) than other countries at the same level of GDP.

**Fig. 5:**
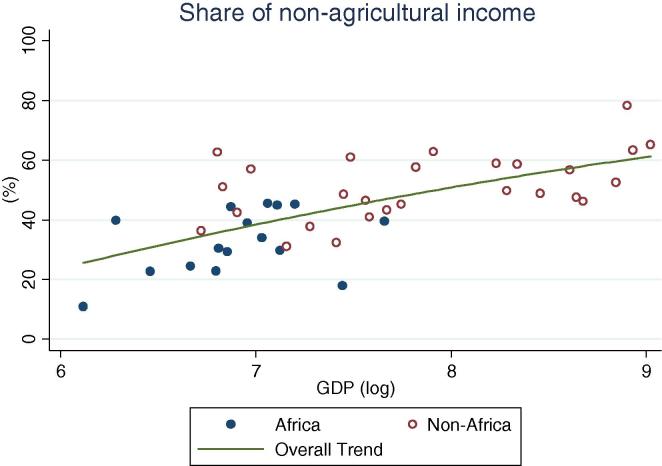
Share of rural households’ non-agricultural income, by per capita GDP in 2005 PPP dollars.

**Fig. 6:**
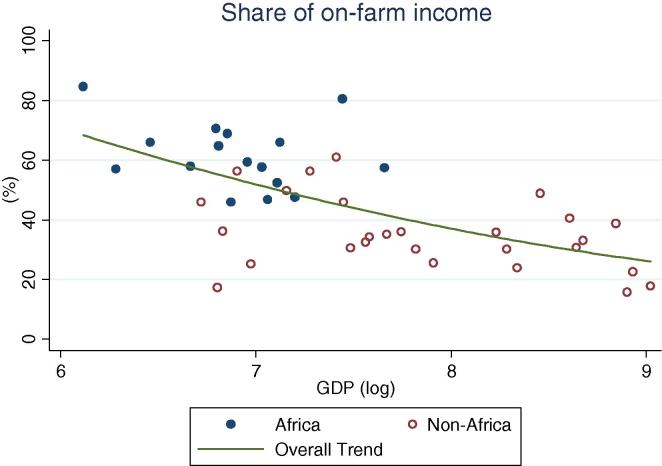
Share of rural households’ on farm income, by per capita GDP in 2005 PPP dollars.

**Fig. 7:**
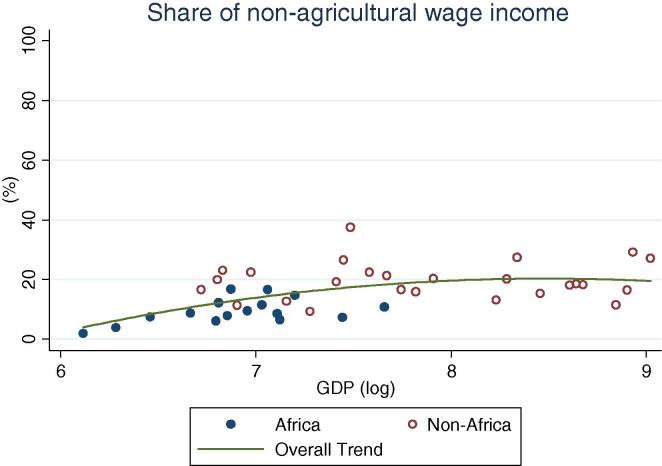
Share of rural households’ non-agricultural wage income, by per capita GDP in 2005 PPP dollars.

**Fig. 8:**
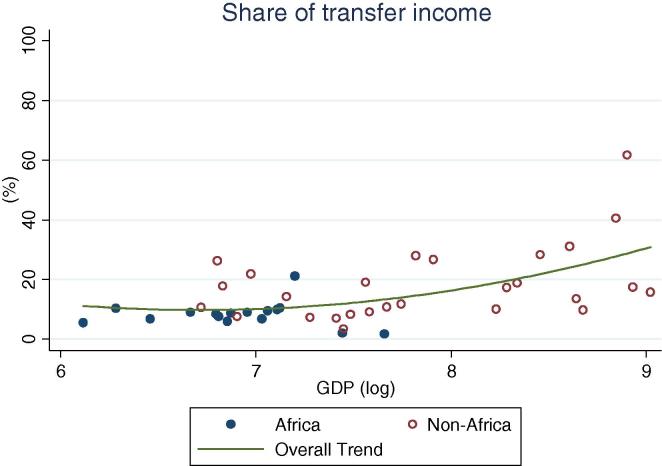
Share of rural households’ transfer income, by per capita GDP in 2005 PPP dollars.

**Fig. 9:**
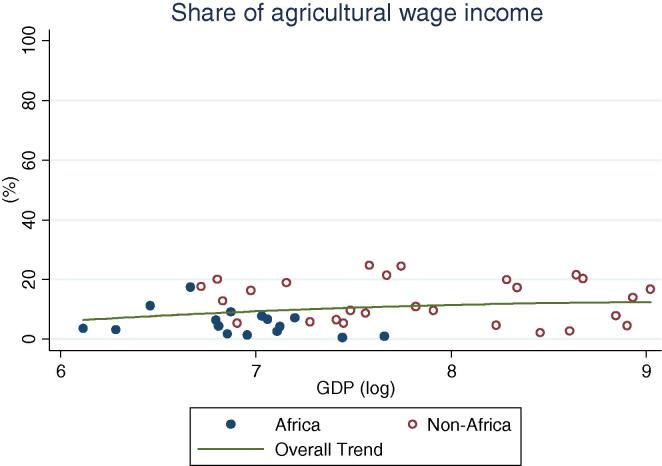
Share of rural households’ agricultural wage income, by per capita GDP in 2005 PPP dollars.

**Fig. 10:**
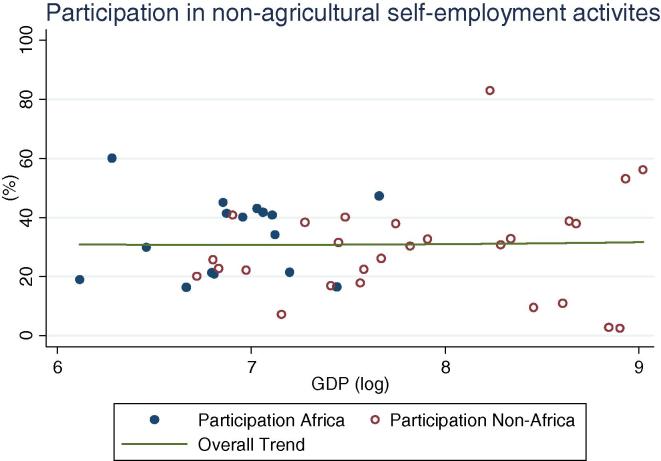
Percentage of rural households participating in no-agricultural self-employment activities, by per capita GDP in 2005 PPP dollars.

Turning to income shares (Table 2, Fig. 5, Fig. 6, Fig. 7, Fig. 8, Fig. 9, Fig. 10), the countries in our African sample show a tendency towards on-farm sources of income (i.e. agricultural income minus agricultural wages): they have higher shares of on-farm income (63 percent) and lower shares of non-farm wage income (8 percent), compared with countries of other regions (33 and 21 percent respectively), including those at similar levels of GDP. All the countries from Sub-Saharan Africa in this sample earn at least 55 percent of their income from agricultural sources, reaching approximately 80 percent in a number of countries (Ethiopia, Madagascar, Malawi, and Nigeria in 2004). Similarly, on-farm income accounts for more than 50 percent in all but one country (Kenya, at 48 percent). Combined with the observation above on the virtually universal level of participation in agricultural activities in the Sub-Saharan Africa subsample, this reinforces the message of agriculture still dominating the rural economy on the continent. Despite the fact that non-agricultural activities are ubiquitous (70 percent participation), they still account on average for only about one third of total earnings.

African countries, particularly those in West Africa, generally have less income from agricultural wage labor (Fig. 9). For Sub-Saharan Africa overall, the maximum share is 15 percent in Malawi; in West Africa, it is a mere 3 percent in Niger. This is an important insight, as some of the expected beneficial effects of high food prices for the poor have been hypothesized to materialize via higher agricultural wages (Ivanic and Martin, 2008). In Africa this is less likely to be the case, compared to countries in Asia and Latin America where agricultural wage income shares in the order of 15–25 percent are far more common. The features of agricultural wage employment are often linked to the peculiarities of the institutions of rural communities (e.g. *ganyu* labor in Malawi), and possibly with the prevalence of plantations and cash crops.

Overall, the share of non-agricultural income among rural households increases with increasing levels of GDP per capita (Fig. 5). The importance of on-farm (crop and livestock) sources of income gradually decreases (Fig. 6) as they are replaced by non-agricultural wage income (Fig. 7) and public and private transfers (Fig. 8). In our sample of African countries, the largest share of income from non-farm sources is recorded in Nigeria (40 percent) and the lowest in Ethiopia (6 percent). Transfer income shares are highest in Kenya (19 percent) and lowest in Nigeria (1 percent), and within this range several countries record substantial shares of 9–10 percent, which is compatible with the documented importance of migrant remittances from urban areas as well as from abroad. Broadly speaking, these values are comparable to the ranges observed in non-African countries.

**Fig. 11:**
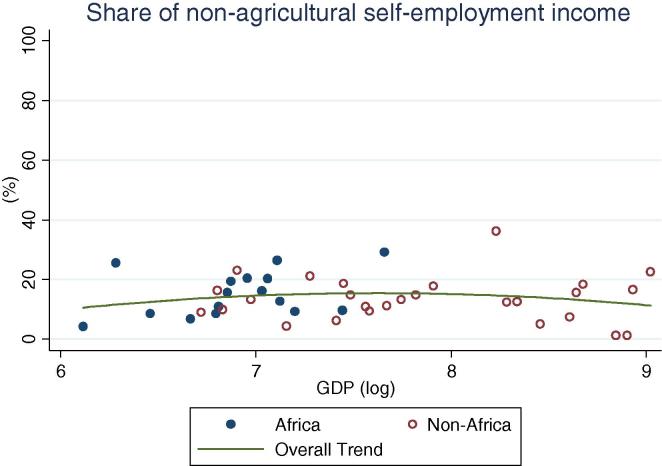
Share of rural households’ non-agricultural self-employment income, by per capita GDP in 2005 PPP dollars.

Lastly, African and non-African countries do not appear to be dissimilar in terms of participation in or shares of income from non-agricultural self-employment (Fig. 10, Fig. 11), where there does not appear to be any clear association with GDP levels.

One important difference between the African and non-African countries in this sample is in the composition of non-agricultural income. While the shares of non-farm self-employment income are comparable across countries in the two groups (14–15 percent), the average share of non-farm wage employment is generally much smaller in SSA, with a maximum level of 15 percent in Kenya in 2005, compared to an average of 21 percent (and peaks of nearly 40 percent) in the non-African component of the sample. This is in line with recent studies of the structural transformation of African economies that have used similar microdata and have found that rural employment in the industry and service sectors is largely in own-account rather than wage occupations, and in services more than in industrial sectors (McCullough, 2015).

### 3.2 Diversification and specialization

The results presented thus far suggest that rural households employ a wide range of income-generating activities, although rural households in African countries are more dependent on agriculture then rural households in other countries. The question remains, however, whether households specialize in activities (with diversity in activities across households in the rural space) or, whether households themselves diversify income-generating activities. If we observe a decline in the share of agricultural income, that could be the result of a few households moving out of agriculture entirely, or of many households marginally reducing their share of income from agriculture.

To explore this question and understand the extent to which households in Africa specialize in agricultural or other sectors relative to households in other regions, we examine the degree of specialization and diversification by defining a household as specialized if it receives more than 75 percent of its income from a single source and diversified if no single source is greater than that amount.^10^^,^^11^

**Fig. 12:**
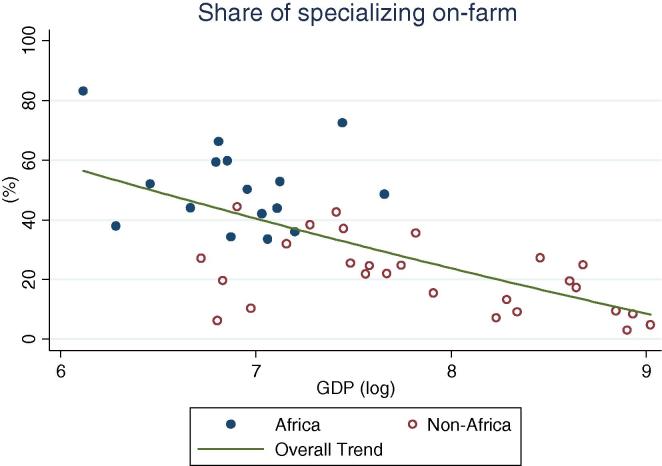
Share of rural households specializing on farm, by per capita GDP in 2005 PPP dollars.

**Fig. 13:**
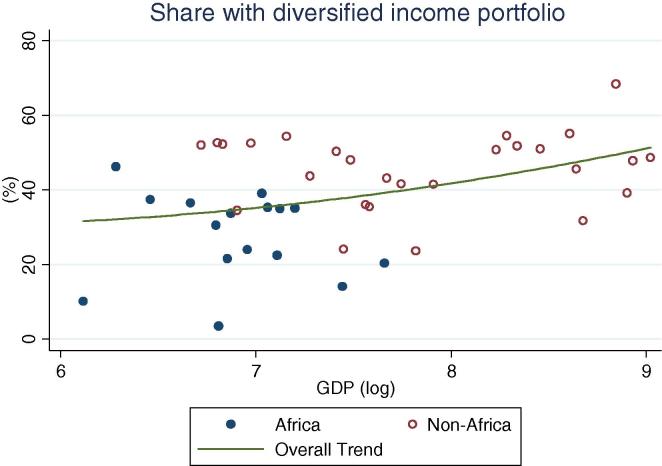
Share of rural households with diversified income portfolio, by per capita GDP in 2005 PPP dollars.

Among rural households in the countries of our African sample, specialization in on-farm activities continues to be the norm (practiced by 52 percent of households on average), ranging from one-third of households in Kenya to 83 percent in Ethiopia (Table 3). Among all countries, with the exception of Niger, a majority of households specialize in on-farm activities. This result is quite different from the non-African households in our sample of countries, where only 21 percent of households on average specialize in farming. Within this group, in only two countries do the majority of households specialize in on farm activities. Diversification is the norm; 45 percent of households fall into the diversified categories, on average. The relative differences between the African and non-African countries with increasing levels of per capita GDP can be seen in Fig. 12, Fig. 13. Rural households in the African country are clustered above the trend line in the former graph, and below the trend line in the latter.

**Fig. 14:**
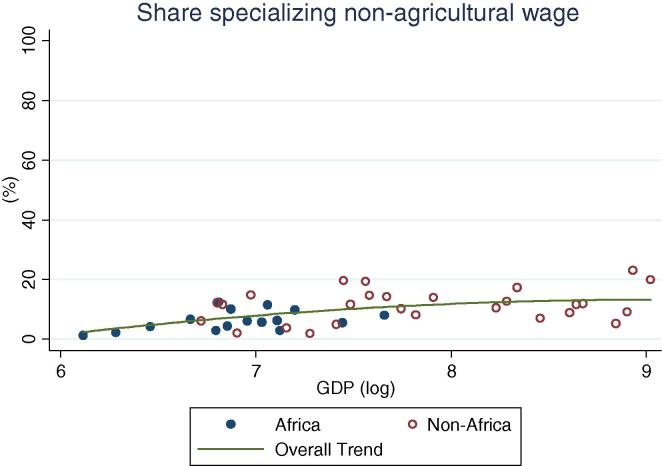
Share of rural households specializing in non-agricultural wage, by per capita GDP in 2005 PPP dollars.

**Fig. 15:**
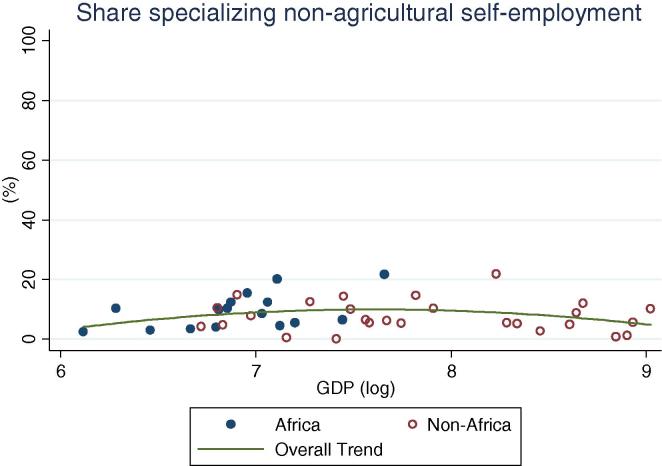
Share of rural households specializing in non-agricultural self-employment, by per capita GDP in 2005 PPP dollars.

When rural households in non-African countries do specialize, they mostly specialize in on-farm activities, although the percentages become lower as the per capita GDP increases. At higher GDP levels, specialization in non-agricultural wage labor becomes more important for both African and non-African countries (Fig. 14). No distinct association between GDP levels and specialization in agricultural wage or self-employment is apparent for non-African countries, while for African countries the share appears to increase (Fig. 15). Taken together, these observations suggest a gradual transition from heavy reliance on farming to a greater reliance on non-farm wage employment, with non-farm self-employment the activity of choice for a more or less constant share of households as development occurs. This essentially confirms the trends observed based on the crude income shares data (Fig. 5, Fig. 6, Fig. 7, Fig. 8, Fig. 9, Fig. 10, Fig. 11 above).

Interestingly, only one of the African countries in our sample has more than 5 percent of households specializing in transfer income (Kenya, with 9 percent). Meanwhile, in non-African countries, it is not at all uncommon for more than 5 percent of households to receive more than three quarters of their earnings from transfers. It is hard to generate robust conclusions from these observations, as transfer income is a mixed bag of several sources (e.g. social protection programs, pensions, migrant remittances, and more) with very different institutional and socio-economic determinants. However, it is worth noting that very few African households are relying mostly on these sources of income for their livelihoods. Despite widespread migration (de Brauw et al., 2014, Ratha et al., 2011) and the expansion of social programs (Garcia and Moore, 2012), productive occupations are what keep most households afloat.

### 3.3 Income sources, returns to different activities, and welfare levels

The previous sections illustrated the diversified nature of the rural economies in all the countries of our sample, including those of Sub-Saharan Africa. Exploring the composition of income at the household level is essential to understanding the strategies and assets that households rely on in order to lift themselves out of poverty. The available literature shows that within both agricultural and non-agricultural income-generating activities, there is often a dualism between high and low return sub-sectors (Nagler and Naudé, 2014). High-return activities often have significant barriers to entry or require accumulation in terms of land, human capital, and other productive assets (Haggblade et al., 2007, Davis et al., 2010). In contrast, a low productivity segment usually serves as a source of residual income or subsistence food production and as a refuge for the rural poor.^12^ Entry barriers may end up confining more marginalized households in low-return sub-sectors, preventing them from taking advantage of the opportunities offered by the more dynamic segments of the rural economy (Reardon et al., 2000). In what follows, our focus will remain at the level of the more aggregated income-generating categories we described earlier, as examining specific industries and occupations is intractable in a cross-country study such as this.

The literature suggests that households participating in higher-return rural non-farm activities are richer and have more upward income mobility (Barrett et al., 2001, Bezu et al., 2012, Bezu and Barrett, 2012, among others), a relationship that holds up in cross country studies and across increasing levels of development (Davis et al., 2010, Winters et al., 2010). Recent studies focus on the dynamics of household participation in rural non-farm activities. Bezu and Barrett (2012) find that households able to accumulate capital, or that have more adult labor or better access to credit and savings, are more able to access high-return rural non-farm activities. Chawanote and Barrett (2013) find the existence of an “occupational ladder” in rural Thailand, in which transitions into the rural non-farm economy lead to increased income, and transitions into farming lead to reduced income. Using data similar to those in our African subset, Nagler and Naudé (2014) find that the productivity of rural household enterprises suffers from the costs associated with large distances, rural isolation, and low population density, and that household enterprises that emerge out of necessity rather than opportunity are systematically less productive.

To explore the relationship across countries between rural income-generating activities and welfare, we start by examining activities by expenditure quintiles for each country. Fig. 16a charts income shares by expenditure quintile for all countries in the African sample. Focusing on on-farm activities, the darkest color, we see a sharp decrease in the share of on-farm income with increasing levels of welfare, dropping from around 50 percent of income in the poorest quintile in most countries, to less than 20 percent in the richest quintile. The drop in on-farm sources of income is made up by the increasing importance of off-farm (i.e. non-agricultural wage and self-employment) sources of income for better-off rural households. The clear trend evident from the countries in the African sample is not as clear in the non-African countries in Fig. 16b. Here Bangladesh, Bulgaria, Nepal, Pakistan and Tajikistan show the opposite trend: the share of on-farm activities increases with welfare.

On the other hand, participation in, and shares of income from, agricultural wage labor show for the most part a negative correlation with the level of expenditure, for both African and non-African countries. With the exception of those countries that have negligible agricultural labor wage markets, poorer rural households tend to have a higher rate of participation in agricultural wage employment. Similarly, the share of income from agricultural wage labor is more important for poorer households in these countries, and the relationship holds regardless of the level of development.

Participation in rural non-farm activities can reflect engagement in either high or low-return sub-sectors. Rural non-farm activities may or may not be countercyclical with agriculture, both within and between years, and particularly if not highly correlated with agriculture, they can serve as a consumption smoothing or risk insurance mechanism. Thus, the results raise the question of whether diversification is a strategy for households to manage risk and overcome market failures, or whether it represents specialization within the household, in which some members participate in certain activities because they have a comparative advantage in those activities. If the latter is the case and it tends to be the young who are involved in off-farm activities, diversification may simply reflect a transition period as the household shifts away from on-farm activities. McCaig and Pavcnik (2014) investigate such an hypothesis for Vietnam and find that less than 20 percent of the shift of labor out of agriculture can be attributed to changing demographics (what they call a between-cohort as opposed to a within-cohort effect).

The empirical relationship between income-generating strategies, diversification and welfare is thus not straightforward. Lower diversification at higher levels of welfare could be a sign that those at lower income levels are using diversification to overcome market imperfections (e.g. cash constraints to finance agriculture, or multiple activities to spread risk). Alternatively, a reduction in diversification at lower income levels could be a sign of an inability to overcome barriers to entry in a second activity, thus indicating that poorer households are limited from further diversification. Higher diversification among richer households could be a sign of using profitability in one activity to overcome threshold barriers to entry in another activity, or complementary use of assets between activities.

The inability to conceptually sign *a priori* the correlation between diversification and household welfare status emerges from the data. Fig. 17a, Fig. 17b explores the relationship between diversification, specialization and household expenditure for the countries in our African sample. The share of rural households with a diversified portfolio of income-generating strategies shows few consistent patterns by quintile of per capita consumption expenditure in our sample countries, in both our African and non-African countries (Fig. 17a, Fig. 17b). A clear pattern emerges, however, among the African countries, in terms of the share of households specializing in on-farm activities. Here, the share of households in most countries decreases with increasing consumption expenditure levels. Conversely, the share of households specializing in self-employment activities and non-agricultural wage labor increases with expenditures, at least for those countries where these activities are prominent, such as Nigeria, Ghana, Malawi and Uganda.

**Fig. 18:**
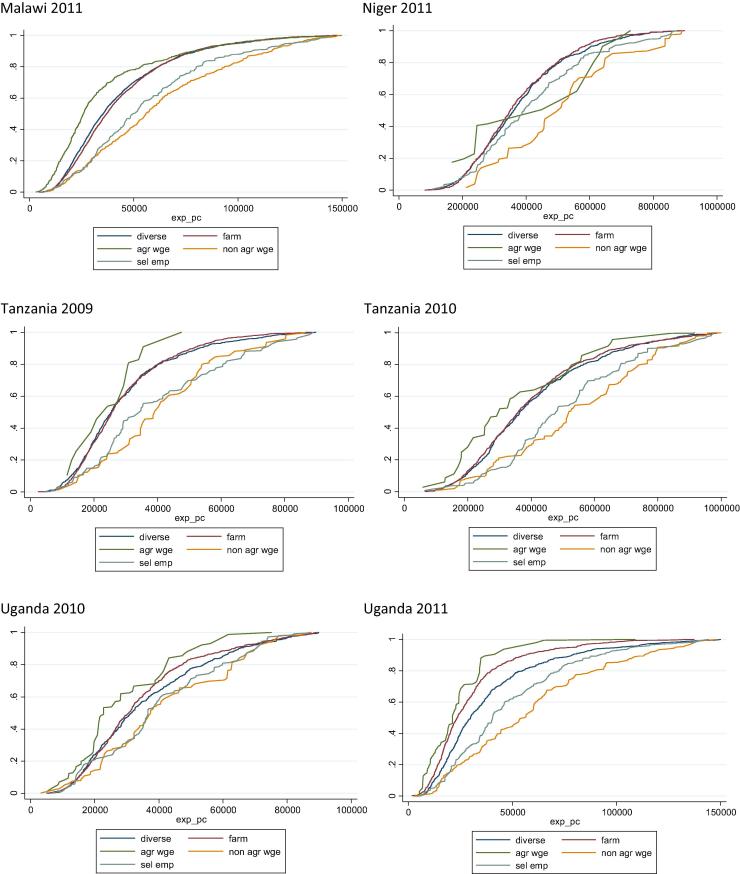
Cumulative per capita expenditure distributions, by income-generating strategy.

Measures of stochastic dominance can complement this analysis by offering a more systematic approach at characterizing the association between household income specialization strategies and the level of household welfare. Stochastic dominance allows for comparing income from different sources and establishing whether one source of income is associated with higher levels of welfare than others. For each of four of the African countries, covering six data sets—Malawi (2011), Niger (2011), Tanzania (2009 and 2010) and Uganda (2010 and 2011)—we plot cumulative density functions (cdf) of consumption expenditures for households in different specialization categories (excluding transfer and other income for clarity of presentation). If cdf lines do not intersect, then we can say that one strategy stochastically dominates another in terms of per capita expenditure (Fig. 18).^13^

Across all countries, specialization in off-farm activities (that is, non-agricultural wage income and self-employment) stochastically dominates other household income-generating strategies, in terms of per capita expenditure (the same analysis, not reported, performed over total household income returns the same ordering). These are followed by on-farm specialization and diversified strategies, and then finally agricultural wage labor which is clearly associated with the lowest levels of welfare.^14^ Overall, these observations confirm the common finding in the literature that increased reliance on non-farm income, particularly in wage employment, is strongly associated with higher levels of overall household welfare, and lower likelihood of being in poverty.

## 4 Modeling location and strategic income choices in LSMS-ISA countries

### 4.1 Estimation approach

As we have noted earlier, much of the literature on rural non-farm income in developing countries has sought to explain how asset endowments and barriers to entry tend to push or pull different households and individuals into different activities. The significance for welfare and poverty analysis and policy has been established in the previous section. Location is an important factor in determining households’ income strategy decisions, but the literature is relatively silent on this point, primarily due to the lack of data that would allow for spatially explicit analysis. The geo-referenced household data that we use makes it possible to begin filling this gap. Since we focus on the rural portion of the sample, we do not discuss issues related to exits from agriculture through household migration to urban areas.

In what follows, our approach is similar to a meta-regression analysis in that: (i) common metrics are used for each of the countries analyzed, (ii) explanatory variables for each country have been created in a uniform manner, and (iii) a standard regression model is employed in each case. This approach minimizes the possibility that differences in results are driven by differences in the variables used or in the empirical approach, and facilitates our comparisons of results across countries.

Our modeling approach is to employ a multinomial logit model (separately for each country) to assess the association of location with the likelihood that a household diversifies or specializes out of farming, controlling for other household characteristics. The choice of the multinomial logit is motivated by the fact that we have several unordered but mutually exclusive categories that we use to characterize household income strategies: a household can either be diversified, or fall within one of six specialization categories.^15^ In the multinomial logit, *k* − 1 models are estimated for any outcome consisting of *k* unordered categories. Parameter estimates are then interpreted with reference to the excluded base category (farm specialization in our case). For a unit change in the regressor, the logit of the model outcome relative to the reference group is expected to change by its parameter estimate, holding other variables constant (UCLA, 2014).

Transforming a continuous variable (income, or income shares which we could have used as the dependent variable) into a categorical one (specialization categories, which is what we use) leads to a loss of information, which should never be taken lightly. In this case, that loss of information is more than compensated for by the fact that using mutually exclusive categories allows us to interpret the data not only in terms of greater or lower involvement in agriculture, but also in terms of the sector towards which households lean as they move away from on-farm specialization. The basic question we aim to address is whether recent growth in rural Africa has been accompanied by less structural transformation of the rural economy than one would expect, given the secular trends observed elsewhere. One advantage of the multinomial logistic regression is that it allows for the use of farm specializers as the reference category. As we use on-farm specialization as the base category, the coefficients on the main variables of interest can be interpreted^16^ in terms of association with higher or lower likelihood that a household specializes in non-farm self-employment, non-farm wage, or agricultural wages relative to specializing in farming. Given the associations noted above between income strategies and welfare, it clearly matters what households do if they do not specialize in farming.^17^ The other advantage is that since we are working with six countries, employing categories that use the same cut-off points increases the comparability of the results.

Previous studies have discussed the role of other key household characteristics, namely different forms of capital (human, natural, physical, social), and these findings are relatively consistent and robust across studies. One concern with that evidence, however, is the extent to which different levels and composition of assets may in fact be endogenous to decisions regarding the income generation strategy. In this paper, the primary interest is to gauge the extent to which truly exogenous factors like climate and distance from urban centers affect household specialization and diversification decisions. Admittedly, distance may itself be endogenous, as existing employment opportunities clearly play a role in a household’s decision on where to live, but we will for convenience put that consideration aside for this discussion. To gauge the effects of distance, market access and agglomeration, we employ the variables described in Section 2 that measure Euclidean distance in kilometers to cities of 20, 100, and 500 thousands inhabitants. For each country regression, we therefore estimate four variants: one per each of the distance variables employed. The reason for differentiating the analysis of distance by city size is linked to the consideration that secondary urban centers offer jobs that demand a different set of skills compared to jobs in large cities, with implications for poverty reduction. Poor rural households with limited human capital may be better able to capture the opportunities offered by secondary towns than those linked to the metropoles or megacities, and the features of the structural transformation of the economy accompanying urbanization may differ depending on whether urbanization is dominated by the expansion of metropoles or accompanied by growth in secondary urban centers (Christiaensen et al., 2013, Hamer and Linn, 1987). Using a cross section of 51 developing country data, Christiaensen et al. (2013, p. 444) find that “only rural diversification and migration to secondary towns is statistically contributing to poverty reduction, while migration to the metropoles is not.”

Agricultural potential is proxied by an aridity index, also described in Section 2 above. To capture the non-linearities in the relationship between specialization/diversification and distance, we introduce both a quadratic term for distance, and interaction terms between distance and aridity. This analysis enables measuring the extent of impact of location effects (i.e. agricultural potential, distance, and their interaction) on the choice of income-generating strategies. In specifying our model using distance to urban centers of different sizes, we are also interested in gauging how these relationships may vary when one considers distance to small towns, as compared to distance to mid-size and large cities.

The vector of regressors includes a range of additional household characteristics that are known to impact decisions about occupational choice and income-generating strategies: separate agricultural and non-agricultural wealth indexes, and an index of access to basic infrastructure (all calculated using principal component analysis); household demographic and composition characteristics (household size, age and gender of the head, number of working age members, share of female working age adults); and variables to measure key households assets (education of the head, land owned).^18^

Based on the theoretical and empirical literature reviewed earlier in this paper, we have some clear expectations as per the sign of the correlation between household endowments and sectors of specialization, with land strongly associated with agricultural activities, education strongly associated with non-farm (particularly) wage activities, and low levels of assets across the board being associated with agricultural wage employment.

**Fig. 19:**
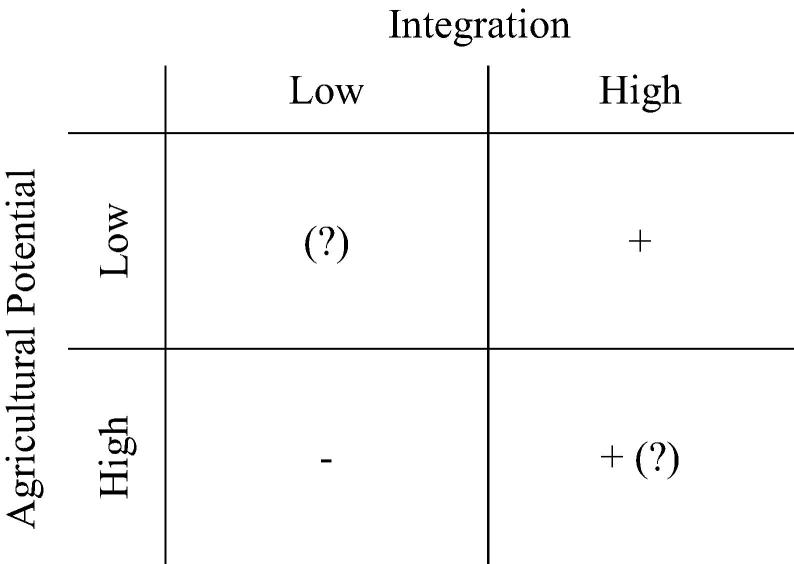
Matrix of expected relationship between specialization in non-agricultural activities, agricultural potential, and integration into urban areas.

To weigh the *a priori* expectations regarding the association between the key location variables (distance and aridity) and diversification or specialization outside of agriculture, we provide a 2 × 2 matrix organized around high/low integration and agricultural potential (Fig. 19).

In high potential, high integration^19^ areas, one expects both farm and non-farm activities to thrive, with non-farm shares dominating as integration levels increase. In low potential, high integration areas, the expectation is for non-farm activities to dominate as people reap off-farm opportunities, as farming does not hold much promise given the unfavorable conditions. Meanwhile, in low integration, high potential areas, the expectation is for farming to be relatively more important. Deichmann et al. (2008) find that in Bangladesh, returns to self and wage employment outside of agriculture tend to decline with distance to the main urban centers, and to decline faster as the agricultural potential increases.

The low-potential low-integration areas are more difficult to sign *a priori*, as on the one hand households will have to rely to a large extent on subsistence farming for their own survival, while on the other hand they will also try to complement the expected meager returns from farming with (possibly equally meager) returns from non-farm activities, including migration. The distinction between diversification from necessity as opposed to from choice proposed by Ellis (2000) is useful in characterizing the situation in these areas.

Our use of a quadratic distance term and of interactions between distance and aridity reflect these expected non-linearities. For the reasons detailed above, the magnitude and signs of these relationships may vary with the size of the urban centers one considers when measuring urban integration.

### 4.2 Results: The impact of distance from urban centers and agricultural potential on household income generation strategies

As summarized in the above discussion, we effectively estimate 5 logit models using 4 different city size categories. We focus the discussion on the extent to which we found presence of non-linearities, their extent and direction, and on the regularities and differences we find across countries, between the role of urban centers of different sizes, and by agricultural potential. To convey the main results emerging from the analysis, we use graphs to demonstrate the broad directions and non-linearities in the main variables of interest (Long and Freese, 2014).

**Fig. 20:**
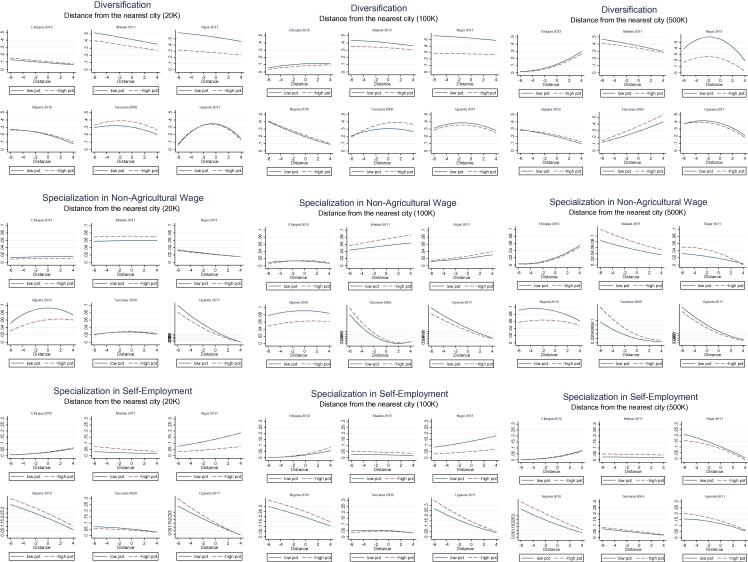
Multinomial logit results: The effect of distance on income strategies, by agricultural potential (aridity) – Base category: Farm specialization.

Fig. 20 reports how the predicted probabilities of being in the diversified and in the main non-farm specialization categories change with distance. To convey the effect of distance separately for high and low potential areas, we graph predicted probability estimated at the 10th (solid line, low potential) and 90th (dashed line, high potential) percentile of the normalized aridity index. The same graphs are reported by distance to cities of different size (20 thousand plus, 100 thousand plus or 500 thousand plus inhabitants). Since one objective of the study is to characterize how (and which) households transition from agriculture to other sectors, we focus on the sectors that identify more engagement in activities outside of agriculture (non-agricultural wage specializers and non-agricultural self-employment specializers), as well as on diversified households, as these constitute a significant share of the total (Table 3). It should be noted that since the sum of the probabilities of households falling into any of the six diversification/specialization categories is equal to one, one should interpret the trends in the three reported categories as the mirror image of the probability of being in one of the other categories, with farming attracting the lion’s share of specializing households (again, refer to Table 3 for the distribution of household into these categories).

The graphs convey the combined effect of the quadratic and interaction terms that are otherwise difficult to interpret from a standard table of coefficients. The first result that emerges is that non-linearities are clearly present in most of the estimated relationships. For most countries and sectors of specialization, the role of distance changes markedly with potential and with city size, but it is difficult to gauge far-reaching regularities. There does not seem to be any universal law governing how the probability of households moving into the non-farm sector varies with distance from urban centers and with agricultural potential. Even within the same country, how the likelihood of households selecting into different categories changes with distance is hardly ever constant across city size or across level of agricultural potential.

To facilitate the interpretation of these graphs, we turn to the relationship between income strategies and distance from cities. As expected, most lines are downward sloping, indicating that the probability of household diversifying or specializing in key non-farm activities declines as the distance from cities increases. There are, however, several exceptions. In Malawi’s low potential areas for instance, the probability of a household being in the diversified category declines from around 50 percent to below 40 percent as distances from towns of 20 thousand plus inhabitants increases. In Niger, a broadly similar trend is observed. Ethiopia and Nigeria also have downward sloping curves, but here the lines for high and low potential areas are virtually overlapping. In Tanzania and Uganda, on the other hand the curves are of an inverted-U shape: they overlap in Uganda, while in Tanzania the probability of being diversified is higher for households in high potential areas at any given distance.

In several cases, the slope of the curves also increases when distance to larger cities is considered, but again, the trend is by no means universal. Specialization in non-agricultural wage in Malawi for instance is rather flat as distance to small towns increases, but clearly downward sloping for cities of half a million people or more. This is consistent with the expectation that larger centers play more of a stimulus factor for non-agricultural occupations, but at the same time we observe cases where the slope is not much affected, or is affected in an opposite direction to what was expected, when the size of the cities being considered increases (e.g. Ethiopia, and Tanzania for self-employment and diversification).

One aspect to note is that the difference in predicted probabilities, whether across high and low potential areas, or over the distance continuum, is often of sizeable magnitude, meaning that understanding these relationships does matter for understanding how these factors play out and interact in shaping household strategies. In Niger, Nigeria and Uganda for instance, the probabilities of specializing in self-employment activities decline by 20–30 points as distance from cities of half a million people or more increases. In Niger, the probability of households diversifying is about twice as large in low potential areas as compared to high potential areas, and differences of similar scale can be observed for non-agricultural wage specialization in Nigeria.

A few considerations can be made when looking at the income generation strategies individually. Diversification, as defined above, is generally more likely close to urban centers, with Tanzania and to some extent Ethiopia being the exceptions. In Tanzania note however the corresponding steep decrease in non-agricultural wage specialization as distance from cities increase, as the two trends are probably two sides of the same story (agricultural wage specialization being replaced by more diversification, a mixed bag of income sources, as distance from cities increases). Where differences in probabilities across high and low potential areas are sizeable (i.e. where the two lines in each graph lie apart), diversification is usually higher in low potential areas (Tanzania being the exception). In Malawi and Niger, the difference in probabilities between low and high potential areas decreases with distance, but does not disappear completely. In Tanzania, where households in high potential areas are more likely to be diversified, the gap with high potential areas increases with distance.

For non-agricultural wage specialization, the probabilities tend to decline with distance in cities of half a million plus, the exception being Ethiopia. For smaller cities the story is mixed, with mostly flat curves when distance from the smaller towns (20 thousand) is considered. There is also a mix of country situations with probabilities of specializing in non-agricultural wage higher in high potential areas in Malawi, Niger, and Tanzania, but lower in Nigeria and Uganda.

For self-employment, the relationship with distance is still present but less generalized. It is consistently downward sloping only in Niger and Uganda, where the levels of specialization in self-employment activities are relatively high near all urban centers. In the other countries it is either flat (Malawi), moderately upward sloping (Ethiopia), or changes from upward to downward sloping as city size increases (Niger). Specialization in self-employment also tends to be more likely in high potential areas in three of the six countries (Malawi, Nigeria, Uganda), whereas the opposite is true in Niger and no difference is observed in Ethiopia and Tanzania (where the levels of self-employment specialization are smallest). Where non-agricultural wage and self-employment specialization probabilities increase with distance, this is usually ‘compensating’ for a decline in diversification from relatively high levels (Malawi, Niger).

We have noted above how the differences by potential (the gap between the two lines in each graph) is sometimes very sizeable, sometimes non-existent. High/low potential areas are associated with different probabilities depending on country, city size and category, with few regularities to speak of. The only country where we never observe a substantial difference between the two ‘strata’ is Ethiopia (note that this is also the country where specialization in farming is dominant in the data) whereas in all other countries the difference matters in at least some of the category/city size combinations. Also, there is a substantial amount of switching of the dominant ‘stratum’ across specialization/diversification categories, less so across city sizes.

These findings speak to different dynamics when the role of small towns is considered and when large cities come into play. For small towns, we find support to the hypothesis that high-potential, low-integration areas see less specialization in off-farm activities, the reverse being true for high-integration low-potential areas. These were the two cells in Fig. 19 for which we had clear *a priori* expectations, but we also found that the role of potential is not particularly strong, at least when the off-farm specialization categories are considered. The two cells where we had unclear expectations were the high potential-high integration, and low-potential low-integration areas. For the former, we find that at least in Tanzania and Uganda the combination of favorable conditions for agriculture and lower distance from urban centers tends to create the conditions for more households to specialize in off-farm activities. When integration is lower and agricultural conditions more difficult, the picture is mixed, with households more likely to engage more fully in non-farm activities in Niger, but less likely to do so in Uganda and Tanzania.

When distance to large cities is considered, the impact of distance is generally more marked, as signaled by the relatively steeper negative slope for both self-employment and non-agricultural wage work. In low-potential, low-integration areas, the sign was uncertain *a priori* and we find that the impact of distance prevails. In high potential areas, we still find the effect of distance generally more than offsetting the effect of potential, which results in decreased odds of being specialized off-farm relative to agriculture as distance from major cities increases. In both cases, Tanzania and Ethiopia counter the trends in at least some of the income categories.

All in all, these results point to evidence that appears to be broadly consistent with the predictions of the theory. There is no sign of African households adopting income generation strategies that differ from those observed elsewhere in terms of their relationship to basic exogenous determinants such as agricultural potential and distance from urban centers. There is however evidence that theory alone cannot be relied upon to predict the net effects of these forces, and that careful, location-specific and spatially explicit diagnostic work is needed to inform policies to facilitate the transformation of rural livelihoods.

## 5 Conclusion

Is Africa’s rural economy transforming as its economies grow? Is it trapped in a growth pattern based on natural resources that may prove unsustainable in the long run? Is there evidence of the share of agriculture in the economy decreasing, following the familiar secular pattern followed by the vast majority of the countries now enjoying middle and high-income status? The analysis in this paper has explored the latest microdata evidence to respond to some of these questions from the perspective of the rural economy.

The analysis of the income-generating activities of rural households based on a large cross-country dataset paints a clear picture of multiple activities across rural space and diversification across rural households. This diversification is true across countries at all levels of development and in all four continents, although less so in the African countries included in the sample. Bearing in mind the caveat that our sample is not representative of the whole of Sub-Saharan Africa, the evidence seems to point towards African patterns of household level income diversification as having the potential to converge towards patterns similar to those observed in other developing regions. While African households are still generally more likely to specialize in farming compared to households in other regions, after controlling for the level of GDP, the shares of income and participation in non-agricultural activities are not dissimilar from those found elsewhere.

For most countries outside Africa (generally with higher levels of GDP), the largest share of income stems from off-farm activities, and the largest share of households have diversified sources of income. However, for the African countries in the sample, most income still derives from on-farm sources. In terms of participation rates, a striking 92 percent of rural households are involved in farming to some extent. Even more remarkably, agricultural income represents 69 percent of total income for the average rural household in Africa, meaning it is by far the most important source of household income. As a result, the median African rural household earns three fourths of its income from agriculture.

Specialization in on-farm income-generating strategies is thus the norm among the African countries in the sample. Agricultural-based sources of income remain critically important for rural livelihoods in all countries, in terms of both the overall share of agriculture in rural incomes and the large share of households that still specialize in agricultural and on-farm sources of income.

While the outcome of a given income-generation strategy will vary by a given household, overall greater reliance on non-farm sources of income is associated with households being richer, in all countries. In almost all cases, better-off households in rural areas have a higher level of participation in (and greater share of income from) non-farm activities. Similarly, richer households have a larger share of specialization into non-agricultural wage employment.

Conversely, agricultural sources of income are generally most important for the poorest households. Income from crop and livestock activities, as well as from agricultural wage labor, represents a higher share of total income for poorer households in almost all countries. Furthermore, a higher share of households specializing in on-farm activities, and particularly agricultural wage employment, is found at the low end of the welfare distribution.

For both African and non-African countries, diversification may function as a household strategy to manage risk and overcome market failures, or represent specialization within the household deriving from individual attributes and comparative advantage. Therefore, diversification can be into either high or low-return sectors, reflect push or pull forces, and represent a pathway out of poverty or a survival strategy.

The results offered here suggest the need to carefully consider how to promote rural development, particularly in Sub-Saharan Africa. Even if development, in the long run, does entail exit from agriculture, the age-old (Johnston and Mellor, 1961) conclusion that this transition needs to happen through investment in the sector, and not its neglect, is still valid today. It is unlikely that inclusive growth and poverty reduction can happen in rural Africa, where half the households specialize in agriculture, without productivity growth in the sector.

The spatial analysis of the factors that drive specialization away from on-farm activities demonstrates that the constraints to off-farm specialization are likely to differ between high- and low-potential and high- and low-integration areas. Additionally, small and large urban centers are likely to exert different influences on the transformation of the rural economy. While this adds complexity to the formulation of policies to promote rural non-farm growth, it also testifies to a series of trends that are not uncommon in other countries, and suggests that after all the African specificity in terms of higher incidence of farming activities may be due more to a GDP-level effect than to a different response by households to the incentives and opportunities coming from agricultural and non-agricultural growth opportunities.

## Figures and Tables

**Table A1:** Countries included in the analysis.

Country	Name of survey	Year collected	Number of observation			Per capita GDP, PPP constant 2005, USD
			Total	Rural	Urban	
*African countries*						
Ethiopia	Rural Socioeconomic Survey	2011/12	3,969	3,969	N/A	454
Ghana	Living Standard Survey	1992	4,552	2,913	1,639	949
Ghana	Living Standard Survey	1998	5,998	3,799	2,199	1,051
Ghana	Living Standard Survey	2005	8,687	5,069	3,618	1,222
Kenya	Integrated Household Budget Survey	2005	13,212	8,487	4,725	1,340
Madagascar	Enquete Permanente Aupres des Menages	1993/94	4,504	2,652	1,852	895
Malawi	Integrated Household Survey	2004/05	11,280	9,840	1,440	640
Malawi	Integrated Household Survey	2010/11	12,271	10,038	2,233	785
Nigeria	Living Standard Survey	2004	17,425	13,634	3,791	1,707
Nigeria	Living Standard Survey	2010	4,682	3,182	1,500	2,120
Niger	Enquête Nationale sur les Conditions de Vie des Ménages et l’Agriculture	2011	3,968	2,430	1,538	535
Uganda	National Household Survey	2005/06	7,424	5,714	1,710	966
Uganda	National Household Survey	2009/06	2,975	2,206	769	1,130
Tanzania	National Panel Survey	2009	3,265	2,063	1,202	1,240

*Non African countries*						
Albania	Living Standards Measurement Study	2002	3,599	1,640	1,959	4,710
Albania	Living Standards Measurement Study	2005	3,640	1,640	2,000	5,463
Bangladesh	Household Income-Expenditure Survey	2000	7,440	5,040	2,400	901
Bangladesh	Household Income-Expenditure Survey	2005	10,080	6,400	3,680	1,068
Bolivia	Encuesta de Hogares	2005	4,086	1,751	2,335	3,758
Bulgaria	Integrated Household Survey	1995	2,468	824	1,664	6,930
Bulgaria	Integrated Household Survey	2001	2,633	877	1,756	7,348
Ecuador	Estudio de Condiciones de Vida	1995	5,810	2,532	3,278	5,658
Ecuador	Estudio de Condiciones de Vida	1998	5,801	2,535	3,266	5,862
Guatemala	Encuesta de Condiciones de Vida	2000	7,276	3,852	3,424	3,966
Guatemala	Encuesta de Condiciones de Vida	2006	13,693	7,878	5,808	4,178
Indonesia	Family Life Survey-Wave 1	1993	7,216	3,786	3,430	2,487
Indonesia	Family Life Survey-Wave 3	2000	10,435	5,410	5,025	2,724
Nepal	Living Standards Survey I	1996	3,370	2,655	715	829
Nepal	Living Standards Survey III	2003	5,071	3,655	1,416	926
Nicaragua	Encuesta de Medición de Niveles de Vida	1998	4,236	1,963	2,273	1,961
Nicaragua	Encuesta de Medición de Niveles de Vida	2001	4,191	1,839	2,352	2,145
Nicaragua	Encuesta de Medición de Niveles de Vida	2005	6,864	3,400	3,464	2,311
Pakistan	Integrated Household Survey	1991	4,792	2,396	2,396	1,719
Pakistan	Integrated Household Survey	2001	15,927	9,978	5,949	1,923
Panama	Encuesta de Niveles de Vida	1997	4,945	2,496	2,449	7,554
Panama	Encuesta de Niveles de Vida	2003	6,363	2,945	3,418	8,267
Tajikistan	Living Standards Survey	2003	4,156	2,640	1,520	1,283
Tajikistan	Living Standards Survey	2007	4,860	3,150	1,710	1,656
Vietnam	Living Standards Survey	1992	4,800	3,840	960	997
Vietnam	Living Standards Survey	1997/98	6,002	4,272	1,730	1,448
Vietnam	Living Standards Survey	2002	29,380	22,621	6,909	1,780

**Table A2:** Participation in income-generating activities by country, rural households.

Country and year	Per capita GDP, PPP constant 2005, USD	Income-generating activity												
		Group I							Group II		Group III			
		(1)	(2)	(3)	(4)	(5)	(6)	(7)	(1) + (2) + (3)	(4) + (5) + (6) + (7)	(1) + (2)	(4) + (5)	(6) + (7)	(3) + (4) + (5) + (6) + (7)
		Agriculture-crops	Agriculture - Livestock	Agricultural wage employment	Non-farm wage employment	Non-farm self-employment	Transfers	Other	*Agricultural total*	*Non-agricultural total*	*On-farm total*	*Non-farm total*	*Transfers & other*	*Off-farm total*
*African countries*														
Ethiopia 2012	454	87%	80%	24%	6%	19%	22%	19%	89%	47%	92%	24%	38%	60%
Ghana 1992	949	87%	54%	4%	14%	45%	37%	6%	*88%*	*73%*	*88%*	*54%*	*40%*	*75%*
Ghana 1998	1,051	88%	51%	4%	18%	40%	41%	13%	*89%*	*75%*	*89%*	*49%*	*49%*	*76%*
Ghana 2005	1,222	85%	43%	4%	13%	41%	36%	4%	*88%*	*69%*	*87%*	*49%*	*38%*	*70%*
Kenya 2005	1,340	89%	79%	13%	25%	21%	53%	13%	*94%*	*74%*	*92%*	*41%*	*57%*	*79%*
Madagascar 1993	895	93%	78%	26%	18%	21%	43%	11%	*96%*	*67%*	*95%*	*36%*	*50%*	*75%*
Malawi 2004	640	96%	65%	55%	16%	30%	89%	7%	*98%*	*93%*	*97%*	*42%*	*90%*	*97%*
Malawi 2011	785	93%	48%	49%	13%	16%	66%	11%	*97%*	*79%*	*93%*	*28%*	*71%*	*91%*
Niger 2011	535	96%	77%	11%	8%	60%	58%	0%	*98%*	*84%*	*98%*	*65%*	*58%*	*86%*
Nigeria 2004	1,707	85%	38%	1%	9%	16%	6%	4%	*86%*	*30%*	*86%*	*24%*	*9%*	*31%*
Nigeria 2010	2,120	81%	53%	3%	14%	47%	3%	5%	*84%*	*57%*	*86%*	*53%*	*8%*	*56%*
Tanzania 2009	1,240	97%	61%	22%	15%	34%	57%	1%	*99%*	*77%*	*98%*	*43%*	*58%*	*82%*
Uganda 2005	966	88%	65%	20%	16%	38%	43%	2%	*92%*	*72%*	*90%*	*49%*	*44%*	*79%*
Uganda 2009	1,130	89%	67%	23%	25%	43%	32%	24%	*92%*	*77%*	*91%*	*56%*	*49%*	*83%*

Simple mean		89%	61%	18%	15%	34%	42%	9%	*92%*	*70%*	*92%*	*44%*	*47%*	*74%*
Minimum		81%	38%	1%	6%	16%	3%	0%	*84%*	*30%*	*86%*	*24%*	*8%*	*31%*
Maximum		97%	80%	55%	25%	60%	89%	24%	*99%*	*93%*	*98%*	*65%*	*90%*	*97%*

*Non African countries*														
Albania 2002	4,710	92%	86%	5%	28%	10%	68%	4%	*93%*	*85%*	*93%*	*35%*	*69%*	*87%*
Albania 2005	5,463	95%	85%	5%	30%	11%	74%	19%	*95%*	*90%*	*95%*	*39%*	*76%*	*92%*
Bangladesh 2000	901	82%	39%	35%	32%	26%	49%	55%	*87%*	*91%*	*79%*	*53%*	*75%*	*97%*
Bangladesh 2005	1,068	85%	73%	29%	35%	22%	42%	59%	*93%*	*90%*	*82%*	*53%*	*76%*	*96%*
Bolivia 2005	3,758	79%	48%	7%	18%	83%	27%	4%	*84%*	*96%*	*81%*	*92%*	*29%*	*98%*
Bulgaria 1995	6,930	65%	41%	22%	37%	4%	66%	14%	*73%*	*86%*	*66%*	*39%*	*69%*	*92%*
Bulgaria 2001	7,348	68%	64%	8%	26%	2%	89%	13%	*78%*	*95%*	*76%*	*29%*	*91%*	*97%*
Ecuador 1995	5,658	74%	76%	39%	34%	39%	27%	48%	*93%*	*85%*	*88%*	*57%*	*62%*	*94%*
Ecuador 1998	5,862	68%	78%	35%	34%	38%	28%	15%	*89%*	*71%*	*85%*	*56%*	*38%*	*86%*
Guatemala 2000	3,966	88%	66%	43%	35%	31%	65%	4%	*93%*	*84%*	*91%*	*53%*	*67%*	*95%*
Guatemala 2006	4,178	81%	46%	31%	51%	33%	71%	3%	*85%*	*90%*	*81%*	*67%*	*72%*	*97%*
Indonesia 1993	2,487	57%	29%	20%	26%	30%	71%	11%	*72%*	*85%*	*61%*	*50%*	*74%*	*89%*
Indonesia 2000	2,724	54%	10%	19%	32%	33%	85%	14%	*64%*	*93%*	*54%*	*55%*	*87%*	*94%*
Nepal 1996	829	93%	82%	42%	35%	20%	26%	8%	*98%*	*69%*	*95%*	*50%*	*32%*	*85%*
Nepal 2003	926	93%	86%	38%	36%	21%	38%	27%	*98%*	*82%*	*96%*	*52%*	*53%*	*91%*
Nicaragua 1998	1,961	71%	68%	42%	38%	22%	33%	4%	*90%*	*67%*	*83%*	*50%*	*36%*	*85%*
Nicaragua 2001	2,145	85%	72%	39%	35%	26%	39%	19%	*95%*	*73%*	*92%*	*52%*	*43%*	*87%*
Nicaragua 2005	2,311	82%	67%	43%	30%	38%	33%	6%	*94%*	*70%*	*90%*	*56%*	*36%*	*84%*
Pakistan 1991	1,719	60%	76%	25%	47%	32%	31%	3%	*84%*	*80%*	*80%*	*68%*	*33%*	*86%*
Pakistan 2001	1,923	40%	65%	20%	48%	18%	31%	16%	*75%*	*78%*	*70%*	*58%*	*41%*	*85%*
Panama 1997	7,554	87%	98%	27%	44%	53%	69%	8%	*99%*	*94%*	*99%*	*79%*	*71%*	*98%*
Panama 2003	8,267	78%	65%	30%	42%	56%	64%	12%	*87%*	*87%*	*82%*	*58%*	*67%*	*94%*
Tajikistan 2003	1,283	89%	69%	49%	29%	3%	58%	1%	*95%*	*73%*	*93%*	*32%*	*58%*	*91%*
Tajikistan 2007	1,656	98%	78%	28%	45%	17%	48%	3%	*99%*	*78%*	*99%*	*56%*	*49%*	*88%*
Vietnam 1992	997	95%	88%	15%	22%	41%	35%	5%	*97%*	*72%*	*94%*	*54%*	*38%*	*77%*
Vietnam 1998	1,448	98%	91%	20%	32%	38%	36%	19%	*99%*	*80%*	*98%*	*59%*	*48%*	*86%*
Vietnam 2002	1,780	79%	68%	11%	39%	40%	83%	25%	*85%*	*96%*	*83%*	*64%*	*87%*	*96%*

Simple mean		79%	67%	27%	35%	29%	51%	16%	89%	83%	85%	54%	58%	91%
Minimum		40%	10%	5%	18%	2%	26%	1%	64%	67%	54%	29%	29%	77%
Maximum		98%	98%	49%	51%	83%	89%	59%	99%	96%	99%	92%	91%	98%

**Table A3:** Share of income-generating activities in total rural household income, by country.

Country and year	Per capita GDP, PPP constant 2005, USD	**Income-generating activity**												
		Group I							Group II		Group III			
		(1)	(2)	(3)	(4)	(5)	(6)	(7)	(1) + (2) + (3)	(4) + (5) + (6) + (7)	(1) + (2)	(4) + (5)	(6) + (7)	(3) + (4) + (5) + (6) + (7)
		Agriculture-Crops	Agriculture - Livestock	Agricultural wage employment	Non-farm wage employment	Non-farm self-employment	Transfers	Other	Agricultural total	*Non-agricultural total*	*On-farm total*	*Non-farm total*	*Transfers & other*	*Off-farm total*
*African countries*														
Ethiopia 2012	454	73%	11%	4%	2%	4%	3%	3%	*88%*	*12%*	*85%*	*6%*	*6%*	*15%*
Ghana 1992	949	66%	3%	2%	8%	16%	6%	0%	*71%*	*29%*	*69%*	*23%*	*6%*	*31%*
Ghana 1998	1,051	55%	4%	1%	10%	21%	9%	1%	*61%*	*39%*	*59%*	*30%*	*9%*	*41%*
Ghana 2005	1,222	49%	3%	3%	9%	26%	10%	0%	*55%*	*45%*	*52%*	*35%*	*10%*	*48%*
Kenya 2005	1,340	32%	16%	7%	15%	9%	19%	2%	*55%*	*45%*	*48%*	*24%*	*21%*	*52%*
Madagascar 1993	895	57%	13%	6%	6%	8%	6%	2%	*77%*	*23%*	*71%*	*15%*	*8%*	*29%*
Malawi 2004	640	56%	9%	11%	7%	9%	6%	0%	*77%*	*23%*	*66%*	*16%*	*7%*	*34%*
Malawi 2011	785	59%	6%	15%	8%	6%	6%	0%	*80%*	*20%*	*65%*	*13%*	*6%*	*35%*
Niger 2011	535	48%	9%	3%	4%	26%	10%	0%	*60%*	*40%*	*57%*	*30%*	*10%*	*43%*
Nigeria 2004	1,707	76%	5%	1%	7%	10%	1%	1%	*81%*	*19%*	*81%*	*17%*	*2%*	*19%*
Nigeria 2010	2,120	48%	9%	1%	11%	29%	0%	1%	*58%*	*42%*	*57%*	*40%*	*2%*	*43%*
Tanzania 2009	1,240	53%	13%	4%	7%	13%	10%	0%	*70%*	*30%*	*66%*	*19%*	*11%*	*34%*
Uganda 2005	966	47%	7%	11%	10%	16%	9%	0%	*65%*	*35%*	*54%*	*26%*	*9%*	*46%*
Uganda 2009	1,130	48%	11%	8%	12%	16%	6%	0%	*66%*	*34%*	*58%*	*28%*	*6%*	*42%*

Simple mean		55%	9%	5%	8%	15%	7%	1%	*69%*	*31%*	*63%*	*23%*	*8%*	*37%*
Minimum		32%	3%	1%	2%	4%	0%	0%	*55%*	*12%*	*48%*	*6%*	*2%*	*15%*
Maximum		76%	16%	15%	15%	29%	19%	3%	*88%*	*45%*	*85%*	*40%*	*21%*	*52%*

*Non African countries*														
Albania 2002	4,710	15%	34%	2%	15%	5%	28%	0%	*51%*	*49%*	*49%*	*21%*	*28%*	*51%*
Albania 2005	5,463	17%	23%	3%	18%	7%	28%	3%	*43%*	*57%*	*41%*	*26%*	*31%*	*59%*
Bangladesh 2000	901	15%	1%	20%	20%	16%	13%	13%	*37%*	*63%*	*17%*	*36%*	*27%*	*83%*
Bangladesh 2005	1,068	18%	9%	16%	22%	13%	9%	12%	*43%*	*57%*	*27%*	*36%*	*21%*	*73%*
Bolivia 2005	3,758	29%	7%	5%	13%	36%	9%	1%	*41%*	*59%*	*36%*	*49%*	*10%*	*64%*
Bulgaria 1995	6,930	13%	8%	13%	24%	2%	37%	2%	*35%*	*65%*	*21%*	*27%*	*39%*	*79%*
Bulgaria 2001	7,348	4%	12%	5%	17%	1%	60%	1%	*20%*	*80%*	*16%*	*18%*	*62%*	*84%*
Ecuador 1995	5,658	9%	3%	10%	39%	23%	9%	6%	*23%*	*77%*	*12%*	*62%*	*15%*	*88%*
Ecuador 1998	5,862	22%	11%	20%	18%	18%	5%	5%	*54%*	*46%*	*33%*	*37%*	*10%*	*67%*
Guatemala 2000	3,966	28%	3%	20%	20%	12%	17%	0%	*50%*	*50%*	*30%*	*33%*	*17%*	*70%*
Guatemala 2006	4,178	21%	3%	17%	27%	13%	18%	0%	*41%*	*59%*	*24%*	*40%*	*19%*	*76%*
Indonesia 1993	2,487	25%	5%	11%	16%	15%	26%	2%	*41%*	*59%*	*30%*	*31%*	*28%*	*70%*
Indonesia 2000	2,724	23%	2%	10%	20%	18%	23%	4%	*35%*	*65%*	*26%*	*38%*	*27%*	*74%*
Nepal 1996	829	32%	14%	18%	17%	9%	10%	1%	*64%*	*36%*	*46%*	*26%*	*11%*	*54%*
Nepal 2003	926	20%	18%	13%	21%	9%	17%	2%	*51%*	*49%*	*38%*	*30%*	*19%*	*62%*
Nicaragua 1998	1,961	23%	11%	25%	23%	9%	8%	1%	*59%*	*41%*	*34%*	*32%*	*9%*	*66%*
Nicaragua 2001	2,145	21%	14%	21%	21%	11%	6%	5%	*57%*	*43%*	*35%*	*32%*	*11%*	*65%*
Nicaragua 2005	2,311	30%	0%	24%	17%	15%	12%	1%	*55%*	*45%*	*30%*	*32%*	*13%*	*70%*
Pakistan 1991	1,719	31%	14%	6%	27%	19%	3%	1%	*51%*	*49%*	*46%*	*45%*	*3%*	*54%*
Pakistan 2001	1,923	21%	11%	9%	29%	11%	15%	5%	*41%*	*59%*	*33%*	*39%*	*19%*	*67%*
Panama 1997	7,554	15%	7%	14%	29%	17%	16%	1%	*37%*	*63%*	*23%*	*46%*	*18%*	*77%*
Panama 2003	8,267	16%	2%	17%	27%	23%	15%	1%	*35%*	*65%*	*18%*	*50%*	*16%*	*82%*
Tajikistan 2003	1,283	37%	17%	17%	12%	1%	15%	0%	*72%*	*28%*	*55%*	*13%*	*16%*	*45%*
Tajikistan 2007	1,656	52%	9%	7%	19%	6%	7%	0%	*68%*	*32%*	*61%*	*26%*	*7%*	*39%*
Vietnam 1992	997	53%	-1%	5%	11%	23%	8%	0%	*57%*	*43%*	*52%*	*34%*	*8%*	*48%*
Vietnam 1998	1,448	41%	15%	6%	9%	21%	7%	0%	*62%*	*38%*	*56%*	*30%*	*7%*	*44%*
Vietnam 2002	1,780	26%	3%	10%	38%	15%	7%	2%	*39%*	*61%*	*29%*	*52%*	*9%*	*71%*

Simple mean		25%	9%	13%	21%	14%	15%	3%	*46%*	*54%*	*33%*	*35%*	*18%*	*67%*
Minimum		4%	-1%	2%	9%	1%	3%	0%	*20%*	*28%*	*12%*	*13%*	*3%*	*39%*
Maximum		53%	23%	25%	39%	36%	60%	13%	*72%*	*80%*	*61%*	*62%*	*62%*	*88%*

**Table A4:** Summary statistics of the variables used in the multinomial logit model.

Variable	Obs	Mean	Std. Dev.
**Ethiopia**			
*Log of euclidean distance 20k*	3,969	3.34	0.80
*Log of euclidean distance 100k*	3,969	4.45	0.81
*Log of euclidean distance 500k*	3,969	5.43	0.56
*Log of euclidean distance 1000k*	3,969	5.51	0.59
*Aridity*	3,969	0.71	0.23
*Ag wealth index*	3,969	0.00	1.00
*Wealth index (non Ag)*	3,969	0.00	1.00
*Infrastructure index*	3,969	0.00	1.00
*Labor force in the HH*	3,969	2.95	1.45
*Female share of labor force*	3,969	0.52	0.31
*Female household head*	3,969	0.22	0.41
*Household head age*	3,969	44.30	15.84
*Household head years of education*	3,969	1.79	3.06
*Household size*	3,969	5.11	2.26
*Land owned, Ha*	3,969	4.09	6.19

Specialization/Diversification			
*Diversified*	3,812	13.72%	0.30
*Specialized in Farm activities*	3,969	68.48%	0.43
*Specialized in Ag wage*	3,812	2.40%	0.10
*Specialized in Non Ag wage*	3,812	3.80%	0.11
*Specialized in Self-employment*	3,969	7.36%	0.15
*Specialized in other income activities*	3,969	4.24%	0.12

**Malawi**			
*Log of Euclidean Distance 20k*	9,816	3.42	0.64
*Log of Euclidean Distance 100k*	9,816	4.08	0.64
*Log of Euclidean Distance 500k*	9,816	4.30	0.83
*Log of Euclidean Distance 1000k*	9,816	6.34	0.14
*Aridity*	9,800	0.68	0.17
*Ag wealth index*	9,816	0.00	0.70
*Wealth index (non Ag)*	9,816	0.00	0.99
*Infrastructure index*	9,816	0.00	1.00
*Labor force in the HH*	9,816	2.11	1.18
*Female share of labor force*	9,816	0.47	0.31
*Female household head*	9,816	0.25	0.43
*Household head age*	9,816	42.89	16.78
*Household head years of education*	9,816	5.35	3.51
*Household size*	9,816	4.60	2.18
*Land owned, Ha*	9,816	0.60	0.50

Specialization/Diversification	9,816		
*Diversified*	9,816	35.92%	0.48
*Specialized in farm activities*	9,816	44.04%	0.50
*Specialized in Ag wage*	9,816	7.78%	0.28
*Specialized in non Ag wage*	9,816	6.69%	0.25
*Specialized in self-employment*	9,816	3.50%	0.18
*Specialized in other income activities*	9,816	2.07%	0.14

**Niger**			
*Log of Euclidean Distance 20k*	2,409	3.79	0.64
*Log of Euclidean Distance 100k*	2,409	4.42	0.59
*Log of Euclidean Distance 500k*	2,409	5.13	0.43
*Log of Euclidean Distance 1000k*	2,409	5.35	0.53
*Aridity*	2,409	0.19	0.05
*Ag wealth index*	2,409	0.00	0.98
*Wealth index (non Ag)*	2,409	0.00	0.94
*Infrastructure index*	2,409	-0.01	0.98
*Labor force in the HH*	2,409	0.96	0.18
*Female share of labor force*	2,409	0.94	0.23
*Female household head*	2,409	0.10	0.30
*Household head age*	2,409	44.61	15.14
*Household head years of education*	2,409	1.37	3.55
*Household size*	2,409	6.42	3.46
*Land owned, Ha*	1,978	4.17	3.88

Specialization/Diversification			
*Diversified*	2,409	46.27%	0.50
*Specialized in farm activities*	2,409	37.90%	0.49
*Specialized in Ag wage*	2,409	0.46%	0.07
*Specialized in non Ag wage*	2,409	2.23%	0.15
*Specialized in self-employment*	2,409	10.47%	0.31
*Specialized in other income activities*	2,409	2.66%	0.16

**Nigeria**			
*Log of Euclidean Distance 20k*	3,177	3.06	1.06
*Log of Euclidean Distance 100k*	3,177	3.58	0.90
*Log of Euclidean Distance 500k*	3,177	4.35	0.79
*Log of Euclidean Distance 1000k*	3,177	4.88	0.71
*Aridity*	3,177	0.83	0.46
*Ag wealth index*	3,177	0.00	1.02
*Wealth index (non Ag)*	3,157	0.00	0.99
*Infrastructure Index*	2,691	0.00	0.96
*Labor force in the HH*	3,177	3.20	2.10
*Female share of labor force*	3,177	0.50	0.27
*Female household head*	3,177	0.14	0.35
*Household head age*	3,169	50.41	15.77
*Household head years of education*	3,102	4.86	5.18
*Household size*	3,177	5.84	3.13
*Land owned, Ha*	3,177	0.49	2.05

Specialization/Diversification			
*Diversified*	3,112	20.33%	0.40
*Specialized in farm activities*	3,112	48.80%	0.50
*Specialized in Ag wage*	3112	0.35%	0.06
*Specialized in NON Ag wage*	3,112	7.88%	0.27
*Specialized in self-employment*	3,112	21.85%	0.41
*Specialized in other income activities*	3,112	0.79%	0.08

**Tanzania**			
*Log of Euclidean Distance 20k*	2,008	3.42	0.86
*Log of Euclidean Distance 100k*	2,008	4.26	0.84
*Log of Euclidean Distance 500k*	2,008	5.51	0.74
*Log of Euclidean Distance 1000k*	2,008	5.90	0.59
*Aridity*	2,008	0.67	0.23
*Ag wealth index*	2,006	0.00	1.01
*Wealth index (non Ag)*	2,006	0.00	1.00
*Infrastructure index*	2,008	-0.13	0.86
*Labor force in the HH*	2,008	2.52	1.56
*Female share of labor force*	2,008	0.51	0.27
*Female household head*	2,008	0.24	0.43
*Household head age*	2,008	47.22	15.92
*Household head years of education*	1,982	4.48	3.40
*Household size*	2,008	5.44	2.92
*Land owned, Ha*	2,008	1.60	1.79

Specialization/Diversification			
*Diversified*	2,008	34.85%	0.48
*Specialized in farm activities*	2,008	52.99%	0.50
*Specialized in Ag wage*	2,008	0.67%	0.08
*Specialized in non Ag wage*	2,008	2.95%	0.17
*Specialized in self-employment*	2,008	4.53%	0.21
*Specialized in other income activities*	2,008	4.02%	0.20

**Uganda**			
*Log of Euclidean Distance 20k*	2,629	2.73	1.14
*Log of Euclidean Distance 100k*	2,629	4.15	1.18
*Log of Euclidean Distance 500k*	2,629	4.72	1.22
*Log of Euclidean Distance 1000k*	2,629	4.83	1.26
*Aridity*	2,605	0.73	0.11
*Ag wealth index*	2,672	0.00	1.66
*Wealth index (non Ag)*	2,688	-0.15	1.10
*Infrastructure index*	2,687	0.00	0.90
*Labor force in the HH*	2,688	2.38	1.49
*Female share of labor force*	2,688	0.50	0.30
*Female household head*	2,688	0.30	0.46
*Household head age*	2,687	43.64	15.90
*Household head years of education*	2,686	4.47	4.04
*Household size*	2,688	5.13	2.88
*Land owned, Ha*	2688	1.65	11.11

Specialization/Diversification			
*Diversified*	2,607	35.39%	0.48
*Specialized in farm activities*	2,607	33.65%	0.47
*Specialized in Ag wage*	2,607	2.81%	0.17
*Specialized in non Ag wage*	2,607	11.61%	0.32
*Specialized in self-employment*	2,607	12.44%	0.33
*Specialized in other income activities*	2,591	4.10%	0.19

**Fig. 16a:**
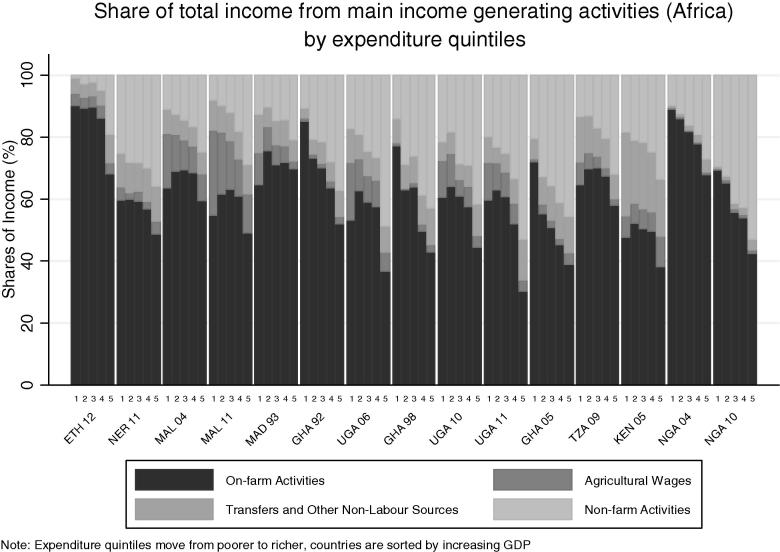
Share of total income from main income generating activities (Africa) by expenditure quintiles.

**Fig. 16b:**
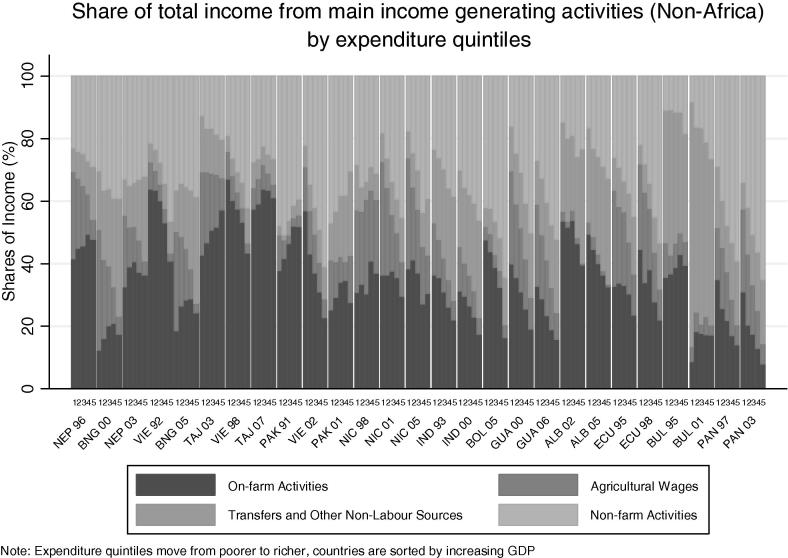
Share of total income from main income generating activities (non-Africa) by expenditure quintiles.

**Fig. 17a:**
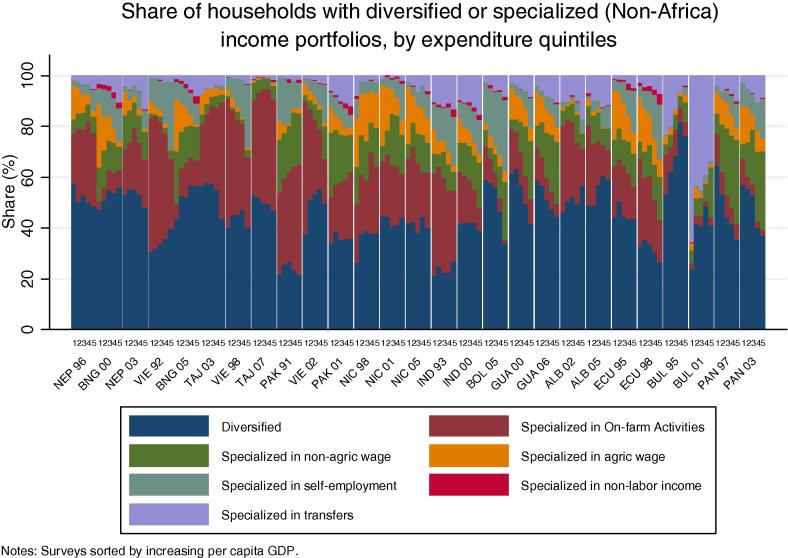
Share of households with diversified or specialized income portfolios, by expenditure quintiles (Non-Africa).

**Fig. 17b:**
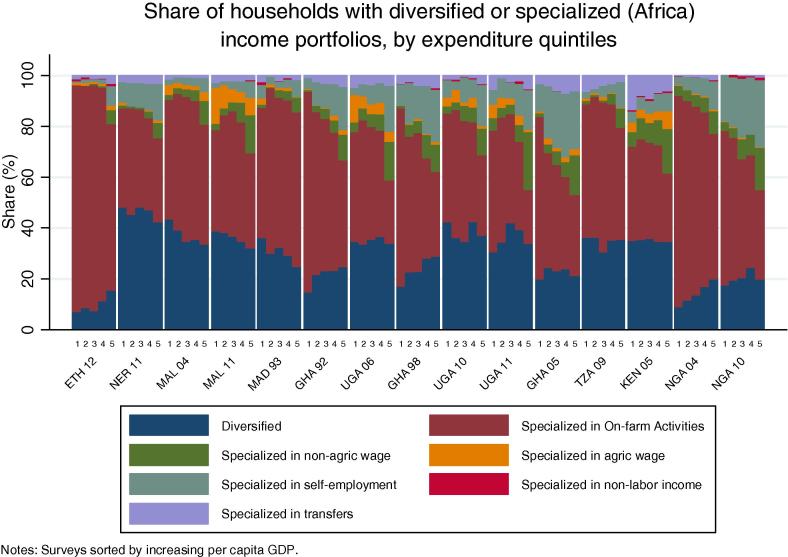
Share of households with diversified or specialized income portfolios, by expenditure quintiles (Africa).

**Table 1:** Participation in income-generating activities, rural households.

		Income-generating activity												
		Group I							Group II		Group III			
		(1)	(2)	(3)	(4)	(5)	(6)	(7)	(1) + (2) + (3)	(4) + (5) + (6) + (7)	(1) + (2)	(4) + (5)	(6) + (7)	(3) + (4) + (5) + (6) + (7)
		Agriculture-crops	Agriculture - Livestock	Agricultural wage employment	Non-farm wage employment	Non-farm self-employment	Transfers	Other	*Agricultural total*	*Non-Agricultural Total*	*On-farm total*	*Non-farm total*	*Transfers & other*	*Off-farm total*
**Africa (14 surveys, 9 countries)**	Simple mean	89%	61%	18%	15%	34%	42%	9%	*92%*	*70%*	*92%*	*44%*	*47%*	*74%*
	Minimum	81%	38%	1%	6%	16%	3%	0%	*84%*	*30%*	*86%*	*24%*	*8%*	*31%*
	Maximum	97%	80%	55%	25%	60%	89%	24%	*99%*	*93%*	*98%*	*65%*	*90%*	*97%*

**Non-Africa (27 surveys, 13 countries)**	Simple mean	79%	67%	27%	35%	29%	51%	16%	89%	83%	85%	54%	58%	91%
	Minimum	40%	10%	5%	18%	2%	26%	1%	64%	67%	54%	29%	29%	77%
	Maximum	98%	98%	49%	51%	83%	89%	59%	99%	96%	99%	92%	91%	98%

*Source*: Authors’ calculations based on the RIGA database. See Table A2 for full results by country.

**Table 2:** Share of income-generating activities in total rural household income.

		Income-generating activity												
		Group I							Group II		Group III			
		(1)	(2)	(3)	(4)	(5)	(6)	(7)	(1) + (2) + (3)	(4) + (5) + (6) + (7)	(1) + (2)	(4) + (5)	(6) + (7)	(3) + (4) + (5) + (6) + (7)
		Agriculture-crops	Agriculture - Livestock	Agricultural wage employment	Non-farm wage employment	Non-farm self-employment	Transfers	Other	*Agricultural total*	*Non-agricultural total*	*On-farm total*	*Non-farm total*	*Transfers & other*	*Off-farm total*
**Africa (14 surveys, 9 countries)**	Simple mean	55%	9%	5%	8%	15%	7%	1%	*69%*	*31%*	*63%*	*23%*	*8%*	*37%*
	Minimum	32%	3%	1%	2%	4%	0%	0%	*55%*	*12%*	*48%*	*6%*	*2%*	*15%*
	Maximum	76%	16%	15%	15%	29%	19%	3%	*88%*	*45%*	*85%*	*40%*	*21%*	*52%*

**Non-Africa (27 surveys, 13 countries)**	Simple mean	25%	9%	13%	21%	14%	15%	3%	*46%*	*54%*	*33%*	*35%*	*18%*	*67%*
	Minimum	4%	-1%	2%	9%	1%	3%	0%	*20%*	*28%*	*12%*	*13%*	*3%*	*39%*
	Maximum	53%	23%	25%	39%	36%	60%	13%	*72%*	*80%*	*61%*	*62%*	*62%*	*88%*

Source: Authors’ calculations based on the RIGA database. See Table A3 for full results by country.

**Table 3:** Percent of rural household with diversified and specialized income-generating activities.

Note: Bordered cells indicate the category with the highest percentage in each country. Shaded cells indicate the specialization category (i.e. excluding diversified) with the highest percentage.

## Appendix

(See Table A1, Table A2, Table A3, Table A4)

## Footnotes

1 See www.worldbank.org/lsms for more information on the LSMS-ISA program of the World Bank, and for full access to the data and documentation.

2 Information on the RIGA database can be found at: http://www.fao.org/economic/riga/en/.

3 Each country has their own definition of rurality, and government definitions not comparable across countries may play some part in explaining cross-country differences. While recognizing that variation in country-specific definitions of rural may explain observed differences in income composition, the available survey data do not allow for straightforward construction of an alternative measure across all countries. We thus use the government definition of what constitutes rurality. Further, rurality is identified via household domicile, not the location of the job – a number of labor activities identified as rural may actually be located in nearby urban areas.

4 Details of the construction of the income aggregates can be found in Carletto et al. (2007).

5 Agricultural income values all production, both consumed on farm and marketed; transfers from both public and private sources (such as remittances) are included; other income covers a variety of non-labor sources of income, such as rental income or interest from savings.

6 The two measures have different meanings. The mean of shares more accurately reflects a household-level income generating strategy, regardless of the magnitude of income. The share of means reflects the importance of a given income source in the aggregate income of rural households in general or for any given group of households. The two measures will give similar results if the distribution of the shares of a given source of income is constant over the income distribution, which is clearly not always the case. If, for example, those households with the highest share of crop income are also the households with the highest quantity of crop income, then the share of agricultural income in total income (over a given group of households) using the share of means will be greater than the share using the mean of shares.

7 CGIAR (2014).

8 A household is considered to participate in an activity if it derives income out of that activity.

9 We considered performing the analysis via a multivariate regression framework, but the sample size is too small.

10 Other definitions of diversification and specialization are possible. Davis et al. (2010) used 100% and 50% of income from a single source as alternative thresholds in order to examine robustness. They find that the extent of diversification is affected by the choice of the threshold, which drops to around 10% or less in all cases when using the 50% definition of specialization, climbing to around 90% with the 100% definition. The broad patterns by country and by level of welfare, however, did not change with choice of the threshold. Alternative groupings of income categories are also possible, such as joining together agricultural and non-agricultural wage labor, or non-agricultural wage labor and non-agricultural self-employment, which would increase the share of household specializing in these new categories.

11 Note that we are constrained from delving into the details of diversification due to the way that household survey data are often collected. The apparent diversification may derive from aggregation across seasons (with seasonal specialization by households) or across individuals (with specialization by individual household members).

12 See Lanjouw and Lanjouw, 2001, Lanjouw and Feder, 2001 for a general discussion relevant to non-farm activities and Fafchamps and Shilpi (2003) for Nepal and Azzarri et al. (2006) for Malawi, for example, regarding the role of agricultural wage labor.

13 We performed pairwise tests of stochastic dominance and they confirm the overall message from Fig. 18 that non-agricultural wage and self-employment specialization tend to stochastically dominate the other income generating strategies. The tests are available from the authors. For interpretation of color in Fig. 18, the reader is referred to the web version of this article.

14 The one exception is specialization in agricultural wage labor in Niger, which includes less than one percent of households, but with relatively high incomes.

15 For the econometric estimation we reduce the specialization categories to five, as we collapse ‘transfers’ and ‘other income’ into one category.

16 With the necessary transformations needed to obtain relative risk ratios.

17 We also experimented with running a similar analysis using a standard OLS regression with the share of income from agriculture as the dependent variable and the results (not reported but available on request) were compatible with those we present below but less informative.

18 Summary statistics for the variables are included in the Appendix Table A4.

19 In what follows, we loosely use the term integration as the inverse of distance.
